# Sorry you asked? *Mayo, Myriad*, and the battles over patent-eligibility

**DOI:** 10.1093/jlb/lsae010

**Published:** 2024-06-04

**Authors:** Robert Cook-Deegan, Janis Geary, Kara Hapke, Zuzana Skvarkova, Marina Filipek, Jillian Leaver

**Affiliations:** Consortium on Science, Policy & Outcomes, Arizona State University; Consortium on Science, Policy & Outcomes, Arizona State University; Consortium on Science, Policy & Outcomes, Arizona State University; Consortium on Science, Policy & Outcomes, Arizona State University; Consortium on Science, Policy & Outcomes, Arizona State University; Consortium on Science, Policy & Outcomes, Arizona State University

**Keywords:** Inherited risk of cancer, patents, molecular diagnostics, genetic testing, coverage and reimbursement

## Abstract

Genetic testing for inherited cancer risk changed dramatically when the US Supreme Court handed down unanimous rulings in *Mayo v. Prometheus* (2012) and *Myriad v. Association for Molecular Pathology* (2013). Those decisions struck down claims to methods based on ‘laws of nature’ (*Mayo*) and DNA molecules corresponding to sequences found in nature (*Myriad*). Senators Thom Tillis (R-NC) and Christopher Coons (D-DE) introduced legislation that would abrogate those decisions and specify narrow statutory exclusions to patent-eligibility in §101 of the US Patent Act. What would be the consequences of doing so? The Supreme Court decisions coincided with changes in how genetic tests were performed, reimbursed and regulated. Multi-gene sequencing supplanted oligo-gene testing as the cost of sequencing dropped 10,000-fold. Payers dramatically changed reimbursement practices. Food and Drug Administration regulation was proposed and remains in prospect. Databases for clinical interpretation made data freely available, augmenting a knowledge commons. The spectacular implosion of Theranos tempered investment in molecular diagnostics. These factors all complicate explanations of why venture capital funding for molecular diagnostics dropped relative to other sectors. Restoring patent-eligibility would put renewed pressure on other patent doctrines, such as obviousness, enablement and written description, that were not raised in the Supreme Court cases.

## I. INTRODUCTION

Four US Supreme Court decisions handed down from 2010 to 2014 changed decades-long understandings about what can be patented in the USA. The decisions directly affected practices in molecular diagnostics. Two decisions—*Mayo v. Prometheus* (2012) and *Myriad v. Association of Molecular Pathology* (2013)—directly addressed diagnostics. They eliminated patent-eligibility for many diagnostic method claims (*Mayo*) and isolated DNA corresponding to sequences found in nature (*Myriad*). This created significant uncertainty about what could be patented in molecular diagnostics (and other fields). The decisions disrupted genetic-testing service monopolies on testing one or a few patented genes for various clinical conditions, including inherited risk of breast and ovarian cancer. Before *Mayo* and *Myriad*, patented genes had been excluded from many tests for the fear of patent infringement liability. The Supreme Court opened the door to widespread, multigene testing by relieving that threat, so formerly patented genes were included in molecular tests, which became available at a lower cost at many more labs.^1^

After the Supreme Court decisions were handed down, several organizations concerned with patent law called upon Congress to abrogate the decisions by revising the definition of patentable subject matter in §101 of the US Patent Act.^2^ These efforts were fueled in part by claims that the decisions dampened investment, and therefore innovation, in clinical genomics. Responding to these claims, Senators Thom Tillis (R-NC) and Chris Coons (D-DE) introduced bills in the US Senate to amend §101 of the Patent Act—most recently S. 2140, the Patent-Eligibility Restoration Act of 2023, which would eliminate judicial exceptions to patent-eligibility and replace them with a limited number of statutory exceptions.^3^

It is unclear whether the bill will become law. At the time of its introduction, GovTrack assessed its odds of enactment at 2 per cent.^4^ Yet, S. 2140 indicates that interest in a legislative remedy to the uncertainty created by patentable subject matter jurisprudence has not dissipated. It is an appropriate time to examine the practical consequences of statutory replacement of Supreme Court decisions with a more permissive doctrine of patent-eligibility. Here, we do so with a focus on consequences for molecular diagnostics, in particular the implications of the *Myriad* and *Mayo* decisions. The paper thus addresses doctrines of patent law but also traces real-world effects on molecular diagnostics.

After providing more detail about the legal background and Congressional response, we turn to the many other factors that changed molecular genetic testing over the same period. We then address some predictable—and some less predictable—consequences if a permissive patent-eligibility statute were to become law.

Our analysis tempers enthusiasm about whether the proposed statutory ‘fix’ would achieve its intended aims of boosting innovation in molecular diagnostics; indeed, it might well hinder it. We do not address other fields affected by current jurisprudence about patentable subject matter.

Patent-eligibility is indeed more uncertain than before the US Supreme Court weighed in. As explained below, however, patent-eligibility is not the only or necessarily even the most important influence on innovation in molecular diagnostics. Moreover, although the Court decisions affected genetic testing, so did many other events that occurred in parallel. The main effect of the Supreme Court decisions was to enable multi-gene testing to include previously patented genes. That is arguably a major innovation in itself.

Even as the Supreme Court was changing patent jurisprudence, DNA sequencing became much faster and its cost dropped 10,000-fold; public databases to interpret the clinical impact of DNA variants were established, making the information much more broadly available to many more institutions; the way genetic tests were coded, billed, covered, and reimbursed changed dramatically; prospects that FDA would directly regulate genetic tests rose and fell episodically, affecting prospective costs of market entry; and the rise and fall of Theranos cast a pall on molecular diagnostics.

These non-patent events confound understanding the significance of the Supreme Court decisions on genetic testing and predicting whether restoring patent-eligibility would improve investment and innovation in molecular diagnostics. Restoring patent-eligibility is unlikely to lead to many, if any, patents on newly discovered human genes in their entirety, however, since there are few left to discover.^5^ Yet, the proposed statutory language would invite patents on methods and on newly discovered variants of known genes and other DNA elements similar to claims granted in the past. Predicting the consequences of restoring patent-eligibility is further complicated if, as is likely, courts would turn to other doctrines of patent law, such as utility (another feature of §101), novelty (§102), obviousness (§103), and enablement and written description (§112). Litigation strategies would change, but courts might well invalidate patent claims using other patent doctrines to reach the same outcomes.

We do not address the wisdom of shifting to a more permissive patentable subject matter jurisprudence in general, but we question whether reverting to the pre-*Mayo* patent-eligibility standards will solve the investment incentive problem it is intended to address. In particular cases, for example, startup firms dependent on venture capital (VC), some molecular tests, and methods might benefit from patent incentives. But, systemwide, returning to pre-*Mayo* patent-eligibility may hinder the improved access to tests and the expanded access and technical innovation that have characterized the past decade of molecular diagnostics.

## II. LEGAL BACKGROUND

### II.A. Patentable Subject Matter at the US Supreme Court

In the past decade, few events have had a more significant impact on US patent law and practice than the US Supreme Court’s four decisions regarding patentable subject matter.

Before these decisions, patentable subject matter was defined largely in the negative: inventions were eligible for patenting unless they were—essentially and when considered as a whole— abstract ideas, laws of nature, or natural phenomena.^6^ Because these exclusions were narrowly defined, inventions were seldom denied a patent based on eligibility, and patents were not often invalidated in court for failure to satisfy the definition of patentable subject matter in §101.

Beginning with *Bilski v. Kappos* in 2010, however, the US Supreme Court transformed the patentable subject matter doctrine from relaxed gatekeeper to vigilant guard. In *Bilski*, the Court held that a test previously established by the US Court of Appeals for the Federal Circuit (the appellate court for all US patent decisions) for determining patentable subject matter—the so-called ‘machine or transformation’ test—provided only an ‘investigative clue’ as to what constituted patentable subject matter, but did not define it.^7^ Then, in 2012, the Court introduced a new patent-eligibility framework in *Mayo* that considered whether claims that recite laws of nature ‘have additional features that provide practical assurance’ that the natural laws are not preempted.^8^ Applying that framework, the Court held that a method of correlating drug effectiveness to metabolite levels of anti-inflammatory drugs recited an ineligible natural law and did not include features sufficient to make it patent-eligible.

A year later in 2013, the Court held in *Myriad* that DNA segments whose sequence is found in nature are ineligible products of nature even if they are isolated.^9^  *Myriad* attracted considerable public attention, and proved highly controversial within the patent bar.^10^

Finally, in 2014 the Court returned to *Mayo’*s eligibility framework in *Alice*,^11^ which the Court described as a ‘minor case’ that did not break new ground but simply followed *Bilski* and *Mayo*. In *Alice*, the Court elaborated that eligibility depends on whether the patent claim at issue is directed to an ineligible concept, and, if so, whether it adds ‘significantly more’ that effectively transforms the claim into a patent-eligible *application* of that concept. Applying this two-step test, the Court held that a method and system for mitigating financial settlement risk were ineligible abstract ideas.

Conventional wisdom about the impact of *Mayo* and *Myriad* on genetic testing holds that ‘The *[Mayo]* ruling opened up the field for broader competition and increased access to genetic testing, as it prevented the monopoly on naturally occurring correlations’ and ‘[*Myriad*] opened up competition and allowed other laboratories to offer testing for *BRCA1* and *BRCA2* genes, leading to increased accessibility and affordability of genetic testing for hereditary breast and ovarian cancer. The ruling also encouraged further research and development in the field, as scientists and companies were no longer restricted by gene patents.’^12^ When introducing nationally prominent genetic counselor Ellen Matloff a decade out, an interviewer observed ‘Few people argue today that the Supreme Court made a mistake.’^13^

Yet, it is not so simple. The people who believe the Supreme Court went astray may or may not be few, but their voices are mighty. As noted below, many experts in patent law are dissatisfied with the current state of the law and have urged Congress to assert its authority to define patentable subject matter and restore patent-eligibility.

### II.B. Subsequent Case Law

The four Supreme Court decisions upended decades of patent practices in biotechnology. Methods and molecules presumed to be patentable since the 1980s were suddenly in question or clearly invalid. However, the impact on patenting in molecular diagnostics was especially pronounced, given the elimination of an entire category of claims on DNA molecules and related methods.

Myriad Genetics’ efforts to enforce claims not challenged in the *Myriad* case proved unsuccessful in litigation after *Myriad* was decided.^14^ Since then, the Federal Circuit has considered a number of diagnostic methods claims under the new patentable subject matter jurisprudence and, to the dismay of some commentators, found them invalid under the *Mayo-Alice* two-step test. In 2015, for instance, the Federal Circuit ruled that a patent on a non-invasive method for detecting fetal abnormalities from DNA was ineligible despite it being a major advance in noninvasive prenatal genetic testing.^15^ The Supreme Court’s decision not to intervene was called the ‘top patent story of 2016,’ as the Court demurred despite pleas from the Appeals Court judges to address the uncertainty surrounding patentable subject matter.^16^ Similarly, in 2018, the same appeals court invalidated a method for detecting *Mycobacterium tuberculosis* by amplifying specific nucleotides.^17^ In 2019, the Federal Court held invalid a test to diagnose risk of cardiovascular disease based on levels of a naturally occurring protein.^18^ Although the Federal Circuit has upheld the patentability of other kinds of method patents—namely, methods of treatment and preparation—‘the bottom line for diagnostic patents,’ concluded one of its judges, ‘is problematic.’^19^

## III. CONGRESSIONAL INTERVENTION

### III.A. Concerns and Backlash

Meanwhile, patent practitioners and legal observers—including David Kappos and Andrei Iancu, two former Directors of the US Patent and Trademark Office (USPTO), as well as prominent members of the patent bar—have turned their attention to Congress. They have weighed in on the impact of the Court’s patentable subject matter decisions on patent practice and innovation and described negative consequences for inventors,^20^ asserting ‘the time has come to amend 35 U.S.C. § 101.’^21^ A retired judge of the Federal Circuit described the current state of patentable subject matter jurisprudence as ‘intolerable chaos.’^22^ Sitting judges concur; the court’s denial of *en banc* review in *Athena Diagnostics, Inc. v. Mayo Collaborative Services, LLC* included an unprecedented eight separate opinions in general agreement on the ‘fraught’ state of ‘the issue of §101 eligibility, especially as applied to medical diagnostics patents.’^23^

We agree there is a problem of uncertainty and inconsistency; we do not, however, find evidence of an innovation deficit for molecular diagnostics that would be addressed by the proposed solution or that expanding patent-eligibility will necessarily improve innovation because of the many other factors that have changed. We explain this position in subsequent sections.

The focus on investment incentives is central because it is the main rationale being proposed for restoring patent-eligibility in the legal scholarship and advocacy reviewed below. Two of the empirical studies point to a reduction in VC funding for molecular diagnostics relative to other fields, and another study finds that small firms have reduced their patent activity in molecular diagnostics. Those studies do suggest a constriction in one pathway to innovation in molecular diagnostics—startup firms securing VC funding based on prospects of patent exclusivity—that was directly affected by the Supreme Court decisions on patentable subject matter.

The empirical studies do not, however, provide evidence of a deficit in overall innovation in molecular diagnostics. Indeed, genetic tests have proliferated over the past decade. Concert Genetics, founded in 2010, tracks clinical genetic testing. Its 2018 ‘landscape’ of genetic testing found over 74,000 tests on the market, which has since increased to 175,000 tests, showing consistent growth in the number of tests year-by-year, and considerable expansion of the market for molecular diagnostics post-*Mayo-Myriad*.^24^ In 2017–2018 alone, 801 multi-gene patent tests were introduced. In the wake of the Supreme Court decisions: ‘Between January 2014 and March 2018, the total number of panels with 3+ genes including *BRCA1/2* grew by more than 10X.’^25^ This expansion of testing came primarily through companies that were already engaged in molecular diagnostics in 2013 and past the stage of VC, or through tests that were developed at academic and hospital laboratories that did not depend on VC funding.

### III.B. Statutory Proposals

In 2017, the USPTO held a workshop to discuss the new jurisprudence responding to concerns about patent-eligibility. In the wake of that workshop, seeking to ‘restore the scope of patent eligible subject matter’ to its *pre-Bilski* state, several professional groups proposed revisions of §101 that would narrowly define exclusions to patent-eligibility.^26^^–^^28^ The recommendations were taken up by Congress in 2019, when Senators Coons (D-DE) and Tillis (R-NC) outlined a proposal to change §101 to ‘statutorily abrogate judicially created exceptions to patent eligible subject matter in favor of exclusive statutory categories of ineligible subject matter.’^29^ The list of exclusions included fundamental scientific principles, mathematical formulae, economic or commercial principles, mental activities, or ‘products that exist solely and exclusively in nature.’ The legislative draft proposal was not translated into legislation that year, but now it has been. On June 22, 2023, Sen. Tillis introduced S. 2140 into the 118th Congress, The Patent-Eligibility Restoration Act of 2023,^30^ a slight revision of S. 4734 from the 117th Congress.^31^ S. 2140 proposes to eliminate judicial exceptions to patentable subject matter and replace them with a limited set of statutory exceptions that include ‘an unmodified human gene, as that gene exists in the human body.’ The Act further clarifies that isolated genes, or genes otherwise altered by human activity, are not ‘unmodified’ and therefore are eligible for patenting. Another provision focused on abrogating *Mayo* and *Alice*, so that ‘any process that cannot be practically performed without the use of a machine (including a computer) or manufacture shall be eligible for patent coverage.’ For molecular diagnostics, the effect of these provisions would be to overturn the *Myriad* and *Mayo* decisions and restore patent-eligibility for both isolated DNA and diagnostic methods.

### III.C. Patent Practices Affecting Genetic Testing for Inherited cancer Risk

Before *Myriad* and *Mayo*, the typical practice in biotechnology patenting was to include claims on the DNA sequence of a gene that had been isolated and characterized in the laboratory, as well as claims on methods for detecting the variants in its sequence associated with a clinical condition or other biological feature. Method and composition of matter claims often resulted in separate patents on the same underlying discovery or invention. The claims that were most difficult to work around for genetic testing were method claims,^32^ most of which were similar to those invalidated by *Mayo* and *Alice*. The composition of matter claims invalidated in *Myriad* were also highly relevant, however, since the process of genetic testing entails making DNA molecules whose sequence mirrors the DNA in a patient’s sample.^33^

### III.D. Sources of Incoherence in Supreme Court Decisions

DNA is both a physical molecule, a chemical, and also the storage and transmission medium for genetic *information*. DNA’s dual nature played a prominent role in the Court decisions in both the USA and a parallel case in Australia. Judge Lourie of the Court of Appeals for the Federal Circuit focused on chemical structure in *Myriad*—he found the breaking of covalent bonds to create a man-made molecule a structural change sufficient to make isolated DNA molecules patent-eligible.^34^ This reversed Judge Robert Sweet’s district court opinion that invalidated all of *Myriad*’s challenged claims. Judge Sweet’s rationale turned on the informational role of DNA, noting that the purpose of a genetic test was to identify the DNA sequence in patients’ cells—that is, to extract information. The use of DNA molecules was merely a means to extract that information.^35^

Justice Thomas’s distinction in *Myriad*, drawing on the arguments presented in *amicus curiae* briefs filed by the Solicitor General,^36^ preserved patent-eligibility for modified DNA—for example, used to make protein therapeutics—while invalidating claims on DNA sequences found in nature. In a separate brief, Eric Lander made that point and added an additional one, directly challenging Judge Lourie’s assertion that DNA fragments of genes with broken covalent bonds are not found in nature. He cited data produced by Christina Fan et al., from the Quake laboratory at Stanford, showing that fetal *BRCA1/2* fragments were detected in a pregnant woman’s blood stream.^37^ These influential briefs were cited by six of the Justices during oral argument.

The Supreme Court echoed Judge Sweet’s informational argument in invalidating the claims pertinent to screening and diagnosis. Yet the Court also implicitly accepted Judge Lourie’s ‘DNA as a chemical’ logic when it noted that cDNA molecules are structurally (chemically) different DNA molecules, making them patent-eligible.^38^ The Supreme Court thus created a rule with a bright-line distinction between what can be patented—an engineered DNA molecule— and what cannot—a DNA molecule whose sequence is found in nature. That incoherence has bred confusion: it implies that to become patent-eligible what matters is changing the structure to something not found in nature, and yet the rationale for invalidation of the diagnostic-relevant claims was DNA’s information content and not its structure.^39^ That is, the rationale for invalidation is based on information content while the justification for patent-eligibility of cDNA is a change in molecular structure. Which is it? The Court drew a bright line between patent-eligible and non-eligible molecules, but left no clue for patent-seekers to predict whether information content or chemical structure should determine patent-eligibility. What should a patent prosecutor advise clients seeking patent protection? If the ‘found in nature’ logic were extended to other molecules, it would eliminate patent-eligibility for valuable therapeutic molecules such as antibiotics and hormones.^40^

Risch and others have argued that novelty, nonobviousness, utility, enablement, and written description could weed out many problematic patent claims, making patentable subject matter dispensable.^41^ Yet, both *Mayo* and *Myriad* rose to the Supreme court as patent-eligibility cases precisely because broad claims survived the prosecution and examination of DNA-based US patents, and litigants chose to focus on patentable subject matter rather than other patent criteria. Perhaps other patent doctrines could be made to do more weeding, but they didn’t.

The inherently dual nature of DNA as the tangible storage and transmission medium for genetic information will continue to present challenges for patent claim interpretation moving forward. A DNA molecule is created in a laboratory whether through genetic testing to determine the DNA sequence in a person’s cells or by using DNA as an intermediary to produce a valuable therapeutic molecule. But those uses are quite different. In genetic testing, any change in the sequence is a mistake that undermines the purpose of testing—to determine the DNA sequence exactly corresponding to that found in the patient’s cells. In producing therapeutics, however, DNA is part of a production chain, and almost always entails deliberate modification of the DNA sequence found in nature, which modification would (probably) make it patent-eligible.

The Supreme Court jurisprudence leaves two unresolved conundrums: (1) a two-step framework for assessing patent-eligibility intended to thwart exclusivity over all uses of fundamental discoveries but also, and probably inadvertently, precluding patents on new diagnostic and screening methods that might benefit from patent incentives; and (2) an inscrutable doctrine that sometimes treats DNA as an information storage medium and sometimes as a chemical, with no discernible consistency.

## IV. NON-PATENT FACTORS AFFECTING EXPECTED PROFITABILITY OF MOLECULAR DIAGNOSTICS

In addition to the shock of lost patent-eligibility for diagnostic uses of gene patents, several other factors significantly changed practices in genetic testing even as *Mayo* and *Myriad* were rising through the courts. The years 2012–2014 saw immense changes in molecular diagnostics, affecting prospects of profitability and incentives for investment and innovation. These factors influence revenues from genetic testing even more directly than patent-eligibility, and thus make it quite difficult to assess the relative importance of patentable subject matter jurisprudence. Coding, billing, coverage, and reimbursement policies for genetic testing would remain in place even if patent-eligibility were restored, casting doubt on whether changing the law would remedy any deficit in investment or innovation incentives.

### IV.A. Hyper-Moore-Curve Drops in the Cost, and Increases in the Speed and Accuracy, of DNA Sequencing

**Figure 1:**
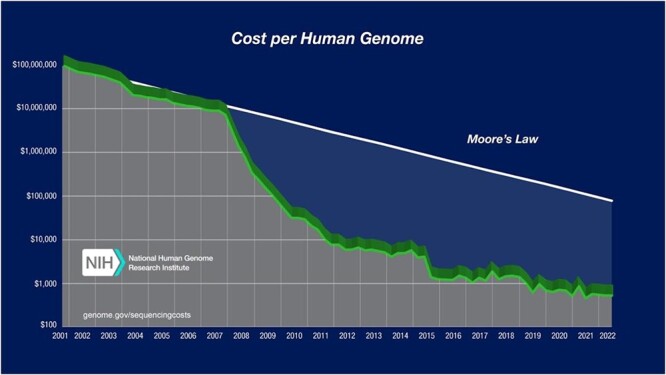
Cost of sequencing a genome. This image copied from https://www.genome.gov/about-genomics/fact-sheets/Sequencing-Human-Genome-cost (accessed March 9, 2024). Generated by Kris Witterstrand of the National Human Genome Research Institute. Not copyrighted.

The most fundamental technical change during this period was the cost of sequencing, which had profound effects on genetic testing. The famous ‘hyper-Moore’s curve’ figure from the National Human Genome Research Institute documents a powerful technological shock, as Sanger sequencing was replaced by next-generation sequence-by-synthesis methods. The sequence-by-synthesis methods were faster while just as accurate. In the decade 2006–2015, the estimated cost of sequencing a human genome dropped 10,000-fold, from ~$12,000,000 to ~$1200 (USD) (see Fig. 1). It is now even lower.

While DNA sequencing costs do not include the interpretation or administrative costs of clinical genetic testing, the sequencing cost is a significant component of DNA testing. From its introduction in 1998 until the Supreme Court decision in 2013, Myriad’s price (not cost) for comprehensive genetic testing *BRCA1/2*—its BRACAnalysis® + BART® tests—rose from $2400 initially to the range of $4100.^42^ That is, the cost of generating a whole genome sequence plummeted from five times more expensive than Myriad’s two-gene *BRCA1/2* test to less than one-third of Myriad’s price over the course of a decade (see Fig. 1). Yet the price of the test rose.

Technological progress meant that labs could generate much more genetic information at lower cost. The drop in sequencing cost led to a shift from testing one or a few genes to sequencing many genes (multi-gene testing), all expressed genes (exome sequencing), or the entire genome (whole-genome sequencing). The technological base of genetic testing shifted from Sanger sequencing of DNA segments amplified by polymerase chain reaction to next-generation genome-wide sequencing. But as Judge Bryson observed in his dissent to the *Myriad* decision at the appeals court level, the genome-wide sequencing strategies infringed claims on individual genes making up the genome.^43^ That is, multi-gene testing could in theory require accumulating rights on all the genes sequenced: the prospect raised by Heller and Eisenberg’s famous ‘anticommons.’^44^

### IV.B. A Pervasive Shift from Testing One or Two Genes to Multi-Gene, Exome, and Whole-Genome Analysis

The shift to widely available multi-gene genetic testing is arguably the most important result of the Supreme Court decisions. Until the *Myriad* decision, few US labs other than Myriad openly performed clinical testing of *BRCA1/2* in the USA for fear of patent infringement liability.^45^ This was also true of other genes subject to patent rights held by other genetic testing labs.^46^^–^^48^

By 1998, Myriad had ‘cleared the market’ of US commercial competitors testing *BRCA1/2* by sending notification and/or cease-and-desist letters, and through two lawsuits that were settled out of court.^49^ Myriad’s US service monopoly on *BRCA1/2* testing evaporated quickly on June 13, 2013, with the Supreme Court decision. Justice Thomas announced the decision just after 10:30 a.m. on June 13, 2013; by noon that day, two firms (Gene by Gene and Ambry) announced they were offering commercial *BRCA1/2* testing. Other firms followed. In July, Myriad initiated lawsuits against commercial competitors, invoking claims that Myriad asserted had not been addressed in the *Myriad* case decided by the Supreme Court. This follow-on litigation did not, however, prevent many other labs from entering the market. By February 2015, those cases had either been dismissed or settled with Myriad signing covenants not to sue for patent infringement.^50^

The *Myriad* decision thus cleared the path not only for *BRCA1/2* two-gene testing, but more importantly, the inclusion of *BRCA1* and *BRCA2* in multi-gene panel testing, as well as the reporting of results of *BRCA1/2* sequences for exome and whole genome analysis. Before June 2013, *BRCA1* and *BRCA2* had been left off cancer multi-gene panels offered by commercial competitors.^51^

After June 2013, other labs added *BRCA1/2* to their panels, offered two-gene testing, or both. As of May 2022, there were 24 companies offering a total of 120 tests for *BRCA1/2* as single genes, and 43 companies offering a total of 211 panels for Hereditary Breast/Breast and Gynecological cancers.^52^ The shift to multi-gene testing was driven by the underlying technology. Instead of stitching together sequences from over 80 ‘amplicons’ (PCR-amplified DNA segments) in the two huge *BRCA1/2* genes, labs developed methods for sequencing many genes associated with inherited cancer risk. The methods differed among laboratories, but the underlying shift to multi-gene testing was pervasive. Even as the *Myriad* case was progressing, Myriad itself was shifting from its two-gene BRACAnalysis® (often augmented by its add-on test for large DNA rearrangements, BART®), to a 25-gene MyRisk® test, which debuted in 2013.^53^^,^^54^ This shift to multi-gene testing would not be possible without licensing a multitude of patents, under the gene-by-gene patent strategies that prevailed in the first generation of gene patents.

### IV.C. A Shift in how Genetic Tests Were Coded for Coverage and Reimbursement

In addition to a shift to multi-gene testing—generating much more genomic information for the same cost—the drop in sequencing cost also elicited a response from institutions that pay for health care, including genetic tests. The process by which genetic testing labs get paid for their service is intricate and complex, which is described in another paper in this special issue.^55^ The Secretary’s Advisory Committee on Genetics, Health and Society described the payment system in a 2006 report, and recommended a more systematic, evidence-based process for deciding on coverage and reimbursement of genetic tests.^56^ The relevance here is that the system for reimbursing genetic tests differs starkly from drugs, biologics, other devices, and other medical goods and services associated with strong profitability. Diagnostics have traditionally been a low-margin business. Myriad’s blockbuster patent-based model was an outlier, not the norm.

For drugs, biologics, and devices (and when Myriad introduced BRACAnalysis®), a manufacturer generally set a price, and then payers responded in a tremendously complicated and opaque process. For most of the period 1998–2012, payment for all genetic tests was based mainly on how many segments of DNA were sequenced. Using the *BRCA1/2* example, Myriad billed for analyzing over 80 amplicons—segments of DNA whose sequences were stitched together by computer to assemble the sequence of both genes.^57^ Tests for other cancer family syndromes, such as Lynch and familial polyposis of the colon, were priced as high or higher on a per-amplicon basis by Myriad and other labs.^58^ The relatively high prices commanded for genetic tests were in part due to Myriad being among the first tests on the market for genetic testing of a relatively common and high-profile genetic condition, and thus being able to argue for its initial $2400 price (and $700 for the follow-on BART® introduced in 2006).^59^ Other labs followed suit. But once in place, reimbursement was formulaic. That is, the Centers for Medicare and Medicaid Services (CMS) paid for lab tests based on the test *method*—usually based on how many DNA segments were sequenced—and it was the same unit reimbursement rate (per DNA segment) for all labs. Private payers used similar payment criteria.^60^

This billing and coding framework changed dramatically in January 2013.^61^ A Clinical Laboratory Fee Schedule was then implemented to pay for genetic tests under Medicare, and other payers used the schedule as a benchmark. The Schedule was based not on method of testing or how many DNA fragments are sequenced, but instead on the clinical condition or the specific gene(s) being tested, with codes assigned to clinical indications. Labs could not set prices for a new product unless it was for an entirely new indication, and they did not control payment levels, because reimbursement was largely determined by the Schedule regularly revised by CMS.^62^ Private payers followed similar practices. That is, through 2012, testing a cluster of genes entailed adding up the number of DNA amplicons for all the genes in a test and sending the bill to payers; it was gene-agnostic. Starting in 2013, it was based on the specific condition or gene(s) being tested.

In a related change, the coding system that undergirds billing was also in flux. The coding system is operated by the American Medical Association.^63^ The codes are used by both federal payers (Medicare, Medicaid, Children’s Health Insurance Program, Veterans Health Administration, the military health system, the Indian Health Service, and others) and private payers (health plans and insurers). Codes determine billing, which, in turn, determines reimbursement. Whether a test is reimbursed is also subject to a determination that it is *covered* by that payer’s plan.

From 2012 to 2014, payers understood DNA testing was being integrated into clinical care. As costs of sequencing dropped, thousands of scientific papers demonstrated associations between genomic variants and disease risk, and new disease-related genes were discovered. Payers became aware that genetic testing could become pervasive and expensive. And expenditures for genetic testing did increase dramatically.^64^ While sequencing costs were plummeting, the costs of encounters with clinicians and interpretation of test results were not; that is, test prices did not drop in parallel to sequencing cost. The prospect of more tests on more people for more conditions loomed as an escalating cost threat to payers. And indeed, by 2021, rising charges to Medicare for genetic testing led the Inspector General to report on the potential for billing scams and unnecessary testing.^65^ Revising the coding system for genetic tests was intended in part to manage an anticipated tsunami of reimbursement claims.

Even as billing codes changed in 2013, the basis for *payment* (pricing) also changed. Section 216 of the Protecting Access to Medicare Act (PAMA) of 2014 established a new framework to pay for clinical laboratory tests.^66^ The CMS (the payer for Medicare, and the federal aspects of Medicaid and Children’s Health Insurance Program) would pay based on a periodically revised schedule based on a survey of laboratories. The selection of labs for the pricing survey was contentious and the subject of litigation, not directly relevant here. But from an investor’s perspective, this PAMA provision was intended to control lab payments and built on the new coding system that linked tests to clinical conditions. The PAMA statute specified implementation in 2016, but it was delayed until 2018. The rolling PAMA payment adjustment scheme was yet another change, layered on top of the new coding and reimbursement system, again well beyond the control of the service providers—the testing laboratories.

Changes in how tests were coded, billed, covered, and reimbursed directly affected revenue streams. Unlike patented drugs, which can be priced by the company introducing a new drug onto the market, diagnostics became reimbursed under a schedule that gave less pricing control to the lab doing the test. The coverage and reimbursement framework is more similar to generic drugs, treated as commodities to be purchased, a contrast with branded and patented new drugs.

Thus, the basis for revenue flows for molecular diagnostics was disrupted at more or less the same time as the Supreme Court was upending patentable subject matter doctrine. This makes coverage, coding, and reimbursement policies crucial elements of innovation policy, arguably more powerful than patent incentives because they are more directly tied to revenues. Indeed, given that reimbursement schedules and payment levels are much easier to tailor and adjust, they may prove both more powerful and more appropriate ways to create incentives for innovation than patent policy, which strives to be agnostic to particular technologies. The fee schedule for genetic tests is, in particular, arguably the most powerful and specific factor affecting incentives to encourage (or discourage) innovation.

### IV.D. Accidental Innovation Policy

While coding, coverage, and reimbursement of genetic tests are powerful drivers of innovation affecting molecular diagnostics, they are generally not framed as innovation policy, but rather as health policy. Conversely, patent policy is generally framed as innovation policy, with little attention to the impact on public health and access. A narrow focus on patents can blind policymakers to other innovation incentives. Several legal scholars have noticed the risk of patent-centric innovation policy. Rachel Sachs reviewed health policies intended to foster access to medical goods and services that nonetheless are powerful influences on innovation, observing health programs are ‘accidental innovation policymakers.’^67^ Lisa Ouellette also pointed to factors beyond patents that are crucial to innovation: research funding, tax incentives, prizes, and data exclusivity; and she cautioned against exclusive focus on patents in innovation policy.^68^ Patents are not the only game in town; indeed for molecular diagnostics, the debate over patent-eligibility may prove a side show compared to coverage and payment policies and regulation: patents may prove important for some specific products, but generally subordinate to the other factors more directly connected to revenue streams: coverage, coding, reimbursement, and regulation. We turn now to regulation.

### IV.E. Prospects of FDA Regulation of Laboratory-Developed Diagnostic Tests

Clinical labs in the US are subject to regulation under the Clinical Laboratory Improvements Act of 1988 (CLIA), which is administered by the CMS. This CMS regulatory function is distinct from its role as a payer for Medicare, Medicaid, and the Children’s Health Insurance Program (CHIP). CMS certifies labs to ensure they comply with quality measures and have sufficient expertise. CMS does not, however, regulate or approve particular tests. Most DNA-based molecular diagnostics have been classified as ‘laboratory-developed’ tests, subject only to CLIA oversight by CMS, not FDA approval. They thus occupy an ambiguous regulatory space. As noted, CMS also *pays* for such tests through Medicare, Medicaid, and CHIP, through its constituent Centers. Thus, CMS is not only the largest single payer, but also the main oversight mechanism for genetic testing.

FDA is not out of the game, however, as prospects of direct FDA regulation of genetic testing have see-sawed for five decades. Test kits that are sold commercially have long been subject to FDA premarket approval under the Medical Device Amendments of 1976.^69^ But FDA practiced ‘enforcement discretion’ regarding laboratory developed tests—tests performed in a laboratory as a service but not sold as a product. FDA asserted it had the authority to regulate genetic tests under the device statute, but chose not to do so. This metastable policy was the subject of considerable debate, dating back to the 1980s. N. Anthony Holtzman wrote an appendix to *Human Gene Therapy*, a 1984 report from the congressional Office of Technology Assessment, that noted the foreseeable rise of genetic testing, and possible need for regulation of genetic tests.^70^ His analysis expanded into a book, *Proceed with Caution*.^71^ The Secretary’s Advisory Committee on Genetic Testing, a federal advisory committee, recommended in 2000 that the ‘FDA should be the federal agency responsible for the review, approval and labeling of all new genetic tests that have moved beyond the basic research phase.’^72^

In 2010, FDA announced an intent to reconsider its enforcement discretion on laboratory-developed tests and hosted a workshop. In 2012, Congress mandated that FDA notify Congress in advance if it intended to regulate laboratory-developed tests. The FDA responded in 2014 with a proposed ‘Framework for Regulatory Oversight of Laboratory Developed Tests.’^73^ The FDA framework elicited considerable push-back from labs and did not yield regulations.^74^ A provision mandating pre-market approval of high-risk molecular diagnostics, aligning with the SACGT recommendation from 2000, was briefly included in drafts of what became the 21st Century Cures Act of 2016.^75^ The diagnostic test regulation provisions were dropped, however, largely because labs, payers, the FDA, and other stakeholders could not reach a consensus. In July 2016, the FDA announced its intent to promulgate regulations, but soon after the 2016 election, withdrew that pledge and instead issued a ‘Discussion Paper’ in February 2017, early in the Trump Administration.^76^

The debate continued into the 117th Congress, with bills introduced into both the House and Senate to authorize FDA regulation as the Verifying Accurate Leading-edge IVCT [*in vitro* clinical test] Development (VALID) Act,^77^ summarized by Rachel Sachs in *Health Affairs*.^78^ A revised version of the VALID bill was tentatively incorporated into pending FDA legislation, and former FDA Commissioners Scott Gottlieb and Mark McClellan urged Congress to pass it.^79^ It nonetheless failed to be included in any legislation passed in the final days of the 117th Congress. Senator Rand Paul also introduced a bill in the 117th Congress, the Verified Innovative Testing in American Laboratories (VITAL) Act, stipulating that ‘no aspects of laboratory-developed testing procedures shall be regulated under the Federal Food, Drug, and Cosmetic Act (21 U.S.C. 301 et seq.).’^80^ The saga continues into the current 118th Congress.

Separately, FDA has the option of pursuing formal regulation under the existing device statute. In June 2023, the Office of Information and Regulatory Affairs in the White House Office of Management and Budget announced the intention to amend FDA regulations pertaining to laboratory-developed tests.^81^ On October 3, 2023, FDA published the notice of proposed rule-making to regulate such tests, and its FDA's rule was announced on April 29 and published on May 6, 2024.^82^ An FDA pilot program also began on June 20, 2023, for diagnostics associated with cancer drugs.^83^ The outcome of FDA regulation is, however, highly uncertain and in any event would take years to roll out.^84^ The new regulation may well be challenged. Molecular diagnostics will continue to be offered under the shadow of regulatory uncertainty.

FDA regulation directly affects the cost of market entry, and thus investment incentives, profitability, and business models. FDA regulation would likely increase barriers to entry, as it does in drugs. Eisenberg reviewed how FDA’s regulatory authority influenced innovation policy in a 2007 review.^85^ Providing the evidence to enter the market would require expenditure on clinical studies. Yet FDA regulation could also become a source of competitive advantage through non-patent mechanisms, such as data exclusivity—that is, barring competitors from using a firm’s data to seek approval of a competing test, thereby enabling protection of R&D investment even without patents.

Hopkins and Hogarth note that FDA regulation might interact with patents on diagnostics similar to features of drug development: patent protection could induce investment in expensive clinical studies of a patent-protected test and preclude free riders from marketing a test without paying for the evidence to prove its clinical utility.^86^ Scholars at the University of Cambridge have also done a series of studies that point to the value, but also the uncertainty, of patent incentives in molecular diagnostics, summarized in 2022.^87^ The implication is that making diagnostic patents more accessible again would increase incentives to invest in clinical research, analogous to the introduction of new drugs.

Payers often already demand evidence of clinical utility to justify coverage and payment, however (at least for major new tests), so the incremental cost of FDA regulation remains unclear. The need for capital to do clinical studies is a major argument for restoring patent-eligibility, but the interdependency between patent rights and incentives for clinical research remain murky. The policy ping-pong of regulating genetic testing, between the FDA and Congress, may well have affected the expected profitability of molecular diagnostics and thus investor enthusiasm for molecular diagnostics—although it is not clear in precisely how, how much, or even in which direction.

Beyond coverage, payment, and regulation, other factors influenced the practice of molecular diagnostics, changing the dynamics of competition and innovation. The next section addresses informational infrastructure that improved the ability of all laboratories throughout the world to interpret the results of genetic tests.

### IV.F. Establishment of Open Data Repositories for Genomic Variants

Genomics as a field evolved a distinctive ‘open science’ ethos, exemplified by the 1996 Bermuda Principles that called for daily disclosure of DNA sequence data from the labs involved in the International Human Genome Sequencing Consortium (the public Human Genome Project, distinct from the privately funded Celera sequencing effort that emerged in 1998).^88^ The Bermuda Principles were developed as patents on *BRCA1* and *BRCA2* were pending. The prospect of patents on individual genes in general, and those two genes in particular, hung like a shadow over the Bermuda proceedings, mentioned frequently by the attendees. Concerns about how patents could impede research were prominent, as detailed by John Sulston in his memoir about the Genome Project, *The Common Thread*.^89^

How patents could affect data flow became apparent during the *Myriad* litigation. As noted in the discussion of proprietary databases above, Myriad stopped sharing most data with public databases in 2004, five years before the litigation began.^90^ Since it had a service monopoly on commercial BRCA testing in the US, Myriad got almost all US samples and therefore was in a unique position to discover new variants in the genes associated with breast and ovarian cancer risk.^91^ Myriad developed a state-of-the-art analytical pipeline for interpreting cancer risk of *BRCA1/2* variants.^92^ Starting in 2004, those data and methods were protected as Myriad’s proprietary assets, under terms of use of their data and web portal.^93^ Clinicians using Myriad’s online system clicked through an agreement to not share data without permission.^94^

### IV.G. Emergence of Public Data Sources for Assessing Genetic Risk

Even as the *Myriad* case was being reviewed by the Supreme Court, a movement to bolster open science in genomics and its applications was taking form. The flood of genomic data emerging from cheaper and faster sequencing caused an explosion of scientific and clinical studies. Genomics needed standards and policies to build a ‘knowledge commons’ to contend with the data deluge.^95^ Fifty colleagues from eight countries met on January 28, 2013, and agreed to produce a white paper that laid out the arguments for a more systematic framework to foster data-sharing.^96^ The white paper proposed a framework, which became the Global Alliance for Genomics and Health, now simply GA4GH.^97^

### IV.H. The Global Alliance for Genomics and Health and BRCA Exchange

One of the three initial flagship projects of the nascent GA4GH was the BRCA Challenge. As *BRCA1/2* testing opened to other labs in June 2013 after *Myriad*, academic labs and Myriad’s commercial competitors had a strong incentive to share data with public databases in order to ‘catch up’ to Myriad’s proprietary database. GA4GH announced the BRCA Challenge to build a data resource for interpreting BRCA variants. Under the Challenge, the BRCA Exchange data resource was established at the University of California Santa Cruz, encouraging data submission from databases and labs around the globe.^98^

While the most common variants in *BRCA1/2* discovered in the early years of testing could be classified as high or low risk, a very large number of rare variants could not be interpreted because the family and clinical case data were insufficient. In 2015, most of those rare variants could be found in only one of the five major databases in the US and Europe.^99^ That is, it required searching five different databases—some of which were only available through subscription—to see if a rare BRCA variant had been reported before, and even then, the large tranche of such variants discovered by Myriad were not available. BRCA Exchange enabled a consolidation. It became a ‘global resource for variants in *BRCA1* and *BRCA2*.’^100^ The first release from BRCA Exchange in October 2016 listed 11,923 variants,^101^ fewer than Myriad had in its proprietary database. By March 2024, BRCA Exchange reported 72,467 variants in *BRCA1/2*.^102^ Since it is freely available on the Internet, this information is available to any clinical laboratory in the world to assist in interpretation.

No one lab, health program, or even national health system has sufficient examples of rare variants to interpret them. Data-pooling is an effort to lay the foundation for a collective knowledge commons, a more effective and efficient way to interpret currently uncertain variants than a congeries of data silos. Myriad’s trade secrecy ironically created incentives for competitor labs to bolster the public data structures collectively, an unusual instance of a multi-player prisoners’ dilemma that resulted in most labs sharing data that could lift all ships.^103^

The emergence of a data commons countervailing against Myriad’s proprietary database meant that Myriad’s competitive advantage would dissipate over time. Investors in the firms contributing to a data commons—Myriad’s competitors—found data-sharing a collective benefit. They competed on other grounds: speed, accuracy, cost, what genes were tested, how results were interpreted, and clarity of reporting. They could also point to the newly open territory after the Supreme Court decisions as an economic opportunity: a market for genetic testing unencumbered by infringement liability. Firms specializing in genomic analysis such as GeneDx, Invitae, Color, and Ambry responded to that opportunity by doing testing and then sharing data with public databases, as a contrast to Myriad. Large, established laboratory firms, such as Quest and LabCorp (both of which started to do BRCA testing after *Myriad*) also entered the market and also shared some variant data with public databases. Such data-sharing was neither universal nor complete, but it was a dramatic enhancement of the public domain resources for genomic interpretation, and data were not constrained by trade secrecy. Most firms that initiated BRCA testing after *Myriad* shared data on variants with BRCA Exchange, and with the newly established database for clinical genetics, ClinVar.

### IV.I. Establishment of the ClinVar Database for Clinical Genomics

The ClinVar database was established at the National Center for Biotechnology Information in 2013, months after *Mayo* and before *Myriad*.^104^ ClinVar was designed to help clinicians to interpret the clinical significance of genomic variants. ClinVar was not established because of the Supreme Court decisions, but its content was bolstered because of them.

ClinVar did not have data on individual cases, which would raise serious privacy concerns if data could be traced to identifiable individuals, but rather listed variants and the risk classifications assigned to them by the labs contributing the data. ClinVar enabled comparisons between conflicting interpretations, and fostered efforts to improve the reliability and accuracy of variant interpretation.^105^^,^^106^ ClinVar quickly became the go-to resource for genetic testing labs all over the world.

In a global survey of clinical testing labs done in collaboration with GA4GH, ClinVar and gnomAD (a population frequency database at the Broad Institute) were the two databases almost universally consulted when making clinical assessments of genomic variants.^107^ ClinVar was for all human genes, not just BRCA or cancer-related genes. ClinVar made it progressively more difficult for labs with proprietary databases to say, ‘Send your samples to us because only we can interpret them.’ The same incentive to build an information commons that gave rise to BRCA Exchange fueled data-sharing with ClinVar, not just for *BRCA1/2*, but also for clinically relevant genes throughout the genome.

### IV.J. The Theranos Effect on Investment in Molecular Diagnostics

Theranos was founded in in California’s Silicon Valley in 2003 by Stanford dropout Elizabeth Holmes. Theranos built on the idea that blood from finger-pricks could quickly and accurately detect health conditions at a low cost. Theranos promised a revolution in laboratory diagnostics. Most of its tests were not genetic tests. Instead, Theranos promised a new wave of inexpensive, readily available common clinical tests. It was not a genetic testing company, but its fate affected perceptions of molecular diagnostics, including genetic testing. Theranos’s valuation rose from $200 million in 2007 to $9 billion in 2014 and transiently exceeded $10 billion.^108^ Yet, by 2018, Theranos was worthless and no longer existed. Its spectacular and highly public disintegration is detailed in *Bad Blood*, by John Carreyrou, the *Wall Street Journal* reporter who broke the story.^109^ Theranos’s demise began in 2015. Theranos raised relatively little of its money from VC firms despite many efforts to do so, with a few exceptions: Tim Draper (Draper Fisher Jurvetson or DFJ), Donald Lucas (Lucas Venture Group or LVG), and Dixon Doll (DCM).^110^ Major pharmaceutical and biotechnology firms also generally demurred. Most of Theranos’s investors were high-wealth individuals such as Betsy DeVos, Henry Kissinger, Rupert Murdoch, and Larry Ellison.

Until 2015, most news was positive and Theranos’s valuation soared. From one perspective, the buzz during Theranos’s rise may have piqued interest in medical diagnostics more generally. The countervailing perspective is that investing in a competitor was betting against a well-funded Silicon Valley firm with an A-list of famous Board members and a compelling storyline, led by a photogenic and charismatic CEO building a global persona. That is, Theranos might have discouraged investment in competitors because of its high public profile and expectations.

As the story of deceit and fraud began to emerge and the firm began to fall apart after October 2015, Theranos became a cautionary tale about the difficulty of medical diagnostics and the perils of investing in them. It ultimately led to prison for founder Elizabeth Holmes and her lover and chief operating officer, Sunny Balwani.^111^^,^^112^ For investors and prospective investors, the billions of dollars that evaporated as Theranos sank beneath the waves cast a pall over molecular diagnostics, breeding caution and skepticism. Steve Brozak in *Stat News* noted ‘The Theranos debacle threatens to make investors even less likely to support diagnostic companies in the future, slowing innovation and delaying the introduction of life-saving tests.’^113^

## V. LEGAL SCHOLARSHIP ON EFFECTS OF PATENT-ELIGIBILITY

Most legal scholarship that attempted to empirically characterize the impact of the four Court decisions on investment in molecular diagnostics did not account for the influence of non-patent factors. David Taylor surveyed 475 VC investors, probing the importance of patent-eligibility in willingness to invest, although at a somewhat less granular level than ‘molecular diagnostics’ and based on survey data. He concluded patent-eligibility was an important (although not always the most important) factor explaining hesitancy to invest in ‘biotechnology, medical device and pharmaceutical industries.’^114^

Olson and Ducci note the potential impact reducing patent-eligibility, and propose several options for re-establishing mechanisms to ensure exclusivity in molecular diagnostics.^115^

**Figure 2:**
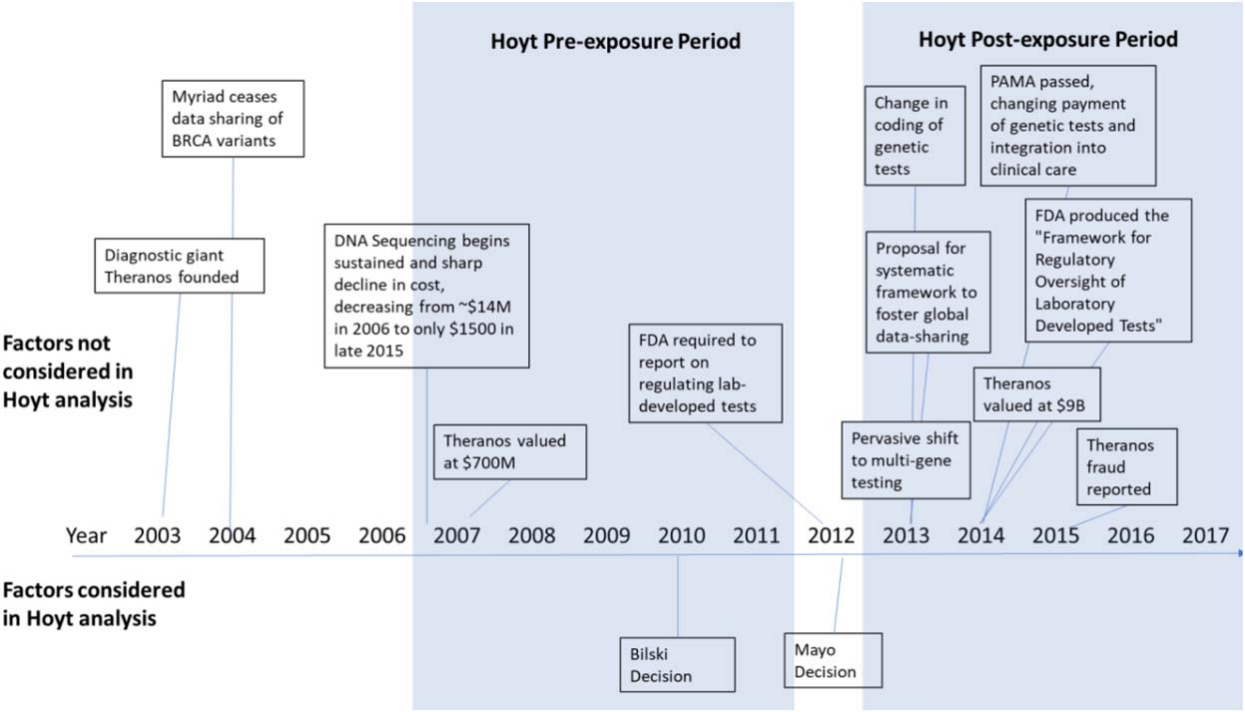
Chronology of events affecting genetic testing for inherited cancer risk. Legend: Timeline of events that influenced VC investments in DNA diagnostics. Supreme Court decisions on patentable subject matter below the line; other notable events above the line.

In another study, Sasha Hoyt compared VC investment in medical diagnostics compared to other sectors using a differences-in-differences analysis, concluding that ‘in the four years following *Mayo*, [venture capital] investment in disease diagnostic technologies was nearly $9.3 billion dollars lower than it would have been absent *Mayo* (Fig. 2).’^116^^,^^117^

Judge Paul Michel, former chief judge of the Court of Appeals for the Federal Circuit, brought a draft of the Hoyt paper to national attention in *Stat News*^118^ arguing that a shortage of Covid tests was due to the Supreme Court’s patentable subject matter decisions. His conclusion: ‘Blame the Supreme Court,’^119^ his remedy: restore patent-eligibility. Judge Michel and coauthors later that year compiled a four-part series presenting evidence for patent-eligibility reform in *IP Watchdog*.^120^^–^^123^

These works are welcome additions to empirical scholarship. But they are all subject to attribution error. They fail to account for the confounds in investment incentives. Instead, they focus exclusively on patents as the only incentives that changed in the multifactorial, storm-torn innovation ecosystem for molecular diagnostics. Patent-eligibility was a factor. It was far from the only factor, however, and arguably not the most important.

Another careful empirical study by Rai, Chien and Clark corroborated some of the findings above. In a sophisticated difference-in-differences analysis, they compared molecular diagnostics to a control set of patents, following their trail through the patent office before and after *Mayo*.^124^ They found that the criteria for getting patents affecting molecular diagnostics became more stringent post-*Mayo*. It took longer from application to patent grant, patents were harder to get (more likely not to survive the process), and the main claims were longer. The length of the primary claim was used as a proxy for breadth of claims, with more words and greater specificity yielding narrower claims. They did not find an overall drop in the patent activity for diagnostics, but they did notice less propensity to patent among small firms, corroborating the findings of Hoyt and Taylor’s VC hesitancy noted above. They were careful, however, not to claim these findings constituted lower innovation in molecular diagnostics overall, noting the many other factors, such as regulation, coverage, and reimbursement were at play.

The interwoven histories of patent-eligibility and other factors are a cautionary tale about exclusively focusing on patents as innovation incentives. Even the baseline assumption of an innovation deficit is arguably wrong, given the well-documented emergence of thousands of genetic tests and market expansion for genetic testing after *Mayo* and *Myriad*.^125^

Two of the most careful and insightful studies of genetic diagnostics and patents were led by Lori Pressman, who was familiar with technology licensing from her previous experience in the Massachusetts Institute of Technology’s licensing office. Pressman first focused on 19 major academic institutions, tracing the rise of US DNA patenting through the 1990s, past its peak in 2001, and pointing to the diversity of uses of such patents: claiming genes that encoded therapeutic proteins, research tools and methods, diagnostics, and other uses. She explored nuances in licensing, with varying degrees of exclusivity and fields of use, and cataloged who owned the patents and the size of firms that licensed them. Her foremost conclusion: ‘Simple reports on exclusive and nonexclusive licensing miss important nuances of licensing practice, nuances that are infrequently discussed in the literature on patenting and licensing.’^126^ Pressman followed this with an analysis of academic institutions, subject to the Bayh-Dole Act,^127^ compared to NIH, which, as a government lab, was instead governed by the Stevenson-Wydler Act.^128^ NIH gave her access to licensing data, which was not available from academic institutions. Under Stevenson-Wydler, the government must advertise availability of an invention and must justify exclusive licensing; nonexclusive licensing is the default. One major conclusion: ‘The field of use in the license agreement is a far superior predictor of the type of commercial product the patent will cover than the patent claims.’^129^ Pressman cashed out her analysis by observing:

‘Exclusivity appropriate to the difficulty of developing, and thus incentives needed to develop diagnostic tests, combined with appropriate diligence requirements... can align interests and maintain flexibility in the dynamic, evolving area of medical diagnostics and personalized medicine.’^130^

Pressman’s second study provides indirect evidence about whether patents stimulate innovation in molecular diagnostics. She found a correlation between patent exclusivity and the speed with which a license generated revenue, suggesting that exclusivity, at least in some cases, can accelerate public availability of a diagnostic test. The evidence is not direct, and her conclusion appropriately tentative, but it is an invitation to research that could find more examples and more details about patent exclusivity and molecular diagnostics by paying close attention to licensing terms. That would, however, require access to licensing data, which are generally not shared by either academic institutions or firms.

In our final section, we also point out—based on literature review, interviews with patent scholars and others,^131^ as well as discussions we have had with lab directors, policy analysts, and advocates—that policy tools other than patentable subject matter might promote innovation in genetic testing for inherited cancer risk more directly and effectively. Our purpose in this final section is not to generate novel policy proposals or extensively review the literature—each section below is or could be the subject of extensive research—but rather to convey the ideas that emerged from talking to experts about how changes in patentable subject matter jurisprudence might affect molecular diagnostics, especially testing for inherited risk of cancer.

## VI. DISCUSSION

### VI.A. A Shift from Injunctive Relief

In the interviews with scholars who had written about patents and molecular diagnostics or those directly engaged in genetic testing, we were surprised to learn that the technological imperative for multi-gene testing was so strong that it might well have emerged despite infringement liability risk, with or without *Mayo* and *Myriad* decisions. The medical director at one lab stated flatly, ‘We were going to do it anyway,’ while acknowledging that it would very likely have entailed a complicated, expensive, and litigious market entry.

It is clear, however, that *Mayo* and *Myriad* dramatically reduced the risk of infringement liability, and made the pathway to multi-gene testing faster, cheaper, and less risky. Several patent scholars we interviewed also observed that patent litigation in the context of multi-gene testing, if it reached the courts, might not warrant injunctive relief. Courts might well search for proportionate remedies. That is, patent-holders would not necessarily be able to block competitors, but might instead get reasonable royalties, based on the strength and breadth of their patent claims and the frequency with which variants in the patented genes were tested and reported back to clinicians and patients. Here, patent scholars point to the Supreme Court decision in *eBay v. MercExchange*,^132^ which unsettled the usual, almost automatic remedy of an injunction when patent infringement was found. Proportionate damages instead of injunctions could prevent a patent thicket from blocking the path to multi-gene or whole-genome testing, but would require a complicated—and likely contentious—process for establishing the functional equivalent of a patent pool or multi-party cross-licensing framework *ex ante*, or for courts to formulae to assess reasonable infringement damages following litigation.

### VI.B. Beyond Patentable Subject Matter

In an attempt to describe the overall landscape that might concern those doing multi-gene testing for inherited cancer risk if patent-eligibility reverted to pre-*Mayo* status, we searched for gene patents whose claims specifically included at least one of the 27 genes tested by all seven of the US clinical labs contributing the most data to the ClinVar database, plus Myriad Genetics.^133^ We identified 3317 granted US patents and 674 published patent applications. As we reviewed claims on some of the most heavily patented genes, we concluded that a careful infringement risk analysis was a large and complicated task that would nonetheless yield uncertain conclusions. We therefore cannot specify the density of the patent thicket, but the large number of patents and complexity of the claims in those patents did make clear that multi-gene testing could incur infringement liability if patent-eligibility rules reverted to the pre-*Mayo*-*Myriad* state. An infringement analysis for a multi-gene, all-exome, or whole genome test would be expensive, laborious, and yet result in considerable uncertainty.

### VI.C. Claims that Could re-Emerge

While the Supreme Court decisions invalidated claims infringed by genetic screening and testing for *BRCA1/2*, not all patent claims were invalidated.^134^ Claims to full-length complementary DNA were explicitly upheld by the Supreme Court. Most methods of genetic testing did not entail making cDNA, however, so preservation of patent-eligibility of modified DNA had little impact on genetic testing. Yet, claims on DNA fragments, primers, probes, and methods that were invalidated by the Supreme Court would be infringed by genetic testing if they became patent-eligible again, and such claims might well resurface if patent-eligibility were restored according to proposed statutory language. Future patents are unlikely to pertain to entire genes because the vast majority of human genes has been disclosed through highly accurate and complete DNA sequencing.^135^ Broad method claims and claims to isolated DNA variants, however, would again be patent-eligible.

Patents granted before *Mayo* and *Myriad* exemplify claims that might once again be granted. US Patent 5753441 claimed any method of detecting a variant in germline testing of *BRCA1*.^136^ That is, discovering the location and sequence of a gene conferred exclusive patent rights to any way of measuring a variant in that gene for the duration of the patent term for any purpose, including research. These and other method claims were judged invalid by the Court of Appeals for the Federal Circuit under the 2012 *Mayo* decision. The broad method claims were thus not addressed by the Supreme Court in *Myriad* because they had already been invalidated by the Appeals Court. Only one method claim survived, on an assay method for screening cancer therapeutic drugs that was irrelevant to genetic testing.

Another example illustrates the potential for patents on variants in known genes. US Patent 7384743, assigned to the University of Miami, claimed ‘A method for analyzing a biological sample from an African American woman for the presence of a polymorphism or mutation associated with breast cancer…’ and listing dozens of specific variants.^137^ This and similar patents on specific variants, or other claims that pertain to only a small fraction of tests, would require a framework for allocating royalties—as noted, a hugely complicated undertaking. This would invite contractual cross-licensing and proportional royalties up front, creating in effect a ‘take but pay’ framework—that is, a liability rule.

Claims like those granted in the past could sprout again for variants in thousands of genes if the proposed statutory language were adopted. There are over 19,000 human genes, with tens of thousands of variants in many of them; vastly more clinically significant variants have yet to be discovered in the human genome. For example, even in *BRCA1/2*—two of the most intensively studied genes in the human genome—many disease-associated variants are predicted but yet to be discovered. While over 72,000 *BRCA1/2* variants have been reported to BRCA Exchange,^138^ an estimated 43–88 per cent (in different sub-populations) of BRCA variants remain to be found.^139^ That proportion of not-yet-discovered variants would be far higher for other human genes less well characterized than *BRCA1/2*.

Case law might accumulate that would invalidate variant-based claims based on obviousness, under *KSR International v. Teleflex* and other cases, or on other grounds.^140^ The Supreme Court’s skepticism about broad molecular claims became apparent in *Amgen v. Sanofi*, using logic that could spill over from claims about monoclonal antibodies to claims about DNA molecules. There, the Court held unanimously that two patents making broad claims to all monoclonal antibodies that inhibited the action of the PCSK9 receptor molecule (to lower cholesterol) were invalid, failing to satisfy enablement under §112.^141^ The analogy is not exact, but courts could find that broad claims to all variants in a gene cannot be claimed without being specifically described, or unless the patent specified some common feature that would distinguish disease-causing from other variants. This would be a high hurdle, since distinguishing disease-associated from benign variants remains a daunting technical and clinical challenge. Case law would have to accumulate for patents claiming DNA variants, based on novelty, non-obviousness, utility, enablement, and written description. Given the dominance of multi-gene panels and the large number of panels being offered, a proliferation of variant-based claims could impact thousands of genetic tests currently on the market. The proposed statutory language would not preclude claims to newly discovered variants, shifting the basis for patent challenges to other patent doctrines beyond §101 patentable subject matter.

### VI.D. Other Concerns with the Proposed Statutory Language

The proposed statutory language precludes patenting ‘an unmodified human gene, as that gene exists in the human body.’^142^ This is ambiguous in several respects. What is ‘human’ DNA? This is not a frivolous question, because patents also apply to nonhuman organisms for many purposes, and include but go well beyond human diagnostic uses. Most DNA sequences trace their origins deep into the roots of the phylogenetic tree; DNA sequences for many genes and other DNA elements are found in nonhuman organisms. Are they patent-eligible?

Among other things, the statutory language would rekindle debates about diagnostic uses of pathogen sequences. It is obvious after the Gallo-Montagnier patent battles over AIDS diagnostics three decades ago, which reached up to the US President and Prime Minister of France, that patents on pathogen diagnostics were valuable, both for saving lives and for making money, as well as achieving scientific stature.^143^

Several decades later, during the Covid pandemic, most home testing for SARS-CoV-19 infection was based on protein testing, but the polymerase chain reaction tests were based on sequences from the virus itself. The *Myriad* decision precluded patenting on DNA molecules whose sequence is found in nature. The proposed statutory language would, however, revive patent-eligibility for pathogen sequences. Indeed, that would be the intent—witness Judge Michel’s argument that Covid diagnostic shortages should be partially blamed on the Supreme Court.^144^ The open question is whether restoring patent-eligibility would foster or hinder diagnostic innovation.

Restoring patent-eligibility for naturally occurring nonhuman DNA would also revive a long-standing debate about whole-genome patenting that was mooted by *Myriad*. In a 2005 review, O’Malley, Bostanci and Calvert identified patents that were based on sequencing entire organisms. They observed:

All the patents we have identified raise important questions about how genomes are conceptualized…. Is the genome just a concept that is used to unite several nucleotide sequences into a single invention, or is it a causally efficacious phenomenon that does something more than an aggregation of genes can do? What is the relationship between the utility of a part (a gene) and any utility associated with the whole (the genome)? The answers to these questions will be different depending on whether the genomes are thought of in terms of biochemistry or bioinformatics… [T]hese patents raise fundamental questions about genome utility, classification, and the ownership of intangible biological information. All these issues mean that the future of genome patenting should be carefully watched by scientists, as much as by legal theorists, social scientists and philosophers of biology—not to mention the patent owners themselves.^145^

Another ambiguity centers on how to interpret ‘as that gene exists in the human body.’ Any diagnostic method or molecule used to extract the information about DNA sequence in the body is not DNA ‘as that gene exists in the body,’ since the diagnostic test DNA is sequenced in a lab, but its value is to derive the sequence as it exists in the body. The apparent intent of the statutory language is to restore patent-eligibility in such cases. But there seems room for interpretation. Would courts agree? That is, does the proposed statutory exclusion mean the exclusion applies to ‘have the same sequence as’ or does it open the door simply to inserting ‘isolated’ into a patent claim to make the method or sequence patent-eligible, standard practice before *Myriad*?

Other legal scholars have argued for changes in the statutory language. Lefstin, Menell and Schmitt render a careful critique of the proposed language. They support S. 2140’s intention to ‘reestablish patent law’s encouragement for scientific breakthroughs and endorse the bill’s abrogation of the *Mayo/Alice* test for patent-eligibility’ but ‘disagree with the bill’s current approach, which ostensibly excludes natural materials and non-technological processes, but then limits those exclusions to the point of insignificance.’^146^

## VII. POSSIBLE REMEDIES

### VII.A. Our Process

In our interviews with 15 patent scholars and experts familiar with molecular diagnostics, we found disagreement about the importance and wisdom of reversing the Supreme Court decisions by statutory definition. There was a strong consensus, however, that the Supreme Court decisions introduced uncertainty, and that it is quite difficult to give advice to clients seeking patents in medical diagnostics. Reducing the incoherence and uncertainty in the Supreme Court decisions would be welcome to all.^147^

The experts pointed to several policies that could address the problems that Judge Shelby identified with the patent-dependent blockbuster model of molecular diagnostics that can ‘hinder or halt follow-up research, data-sharing, patient testing, and the creation of additional and more affordable technologies… [and] turns much of our patent system policy on its head.’^148^ Many of the policy options deal either with patent doctrines other than patentable subject matter, carve out activities that would be exempt from infringement liability, or address remedies for infringement.

We briefly summarize some of these options. The purpose is not to review the literature or exhaustively survey policy options, but rather to convey ideas that emerged from talking to those directly familiar with the intersection of patents and genetic diagnostics.

### VII.B. Raise the Standard for Obviousness

Building on *KSR*, examiners could make the size of the ‘inventive step’ bigger than simply discovering a new variant. This could thin the thicket of claims by focusing on the nature of the invention.

### VII.C. Raise the Threshold for Enablement, Written Description and Definiteness

This option focuses on the specification and claims of the patent document itself, not on the nature of the invention. These patent doctrines—each of which has a body of case law and legal scholarship to guide interpretation—arise from §112 of the Patent Act. §112 can thus be a tool for constraining claims to ensure that the degree of exclusivity is proportional to the disclosure. Narrow claims are weaker because they are easier to work around and cover fewer potential future competing products and services, but narrow claims can also prove more enforceable, more robust in litigation and reduce the risk of pre-empting follow-on research and innovation.

Elevating the threshold for patentability under §112 would come with some disadvantages. One is the higher cost of fact-intensive litigation and discovery required for §112 litigation compared to summary judgments based on §101 patentable subject matter determinations. But as noted above, raising the threshold for §112 was the basis for the 2023 decision in *Amgen v. Sanofi*,^149^ suggesting the Supreme Court might be inclined to make criteria for DNA-based patents under §112 more stringent if Congress reins back patent-eligibility to pre-*Mayo* status.

### VII.D. Establish a Framework for Proportionate Damages as an Alternative to Injunctive Relief

Case law could develop a framework for apportioning damages in cases where infringement is found. This would reduce the value of patents that pertain to genes that are less often tested or less frequently associated with disease risk. And it could also apply to claims on new variants discovered in genes, analogous to the Miami patent cited above.^150^ The prospect is daunting, however, given the profusion of genetic tests and the accounting procedures that would be involved.

Sorting through even just the 3991 US patents and applications with claims citing the 27 most tested genes conferring inherited cancer risk proved overwhelming in our partial patent landscape, yet most cancer panels are now larger than 27 genes, and the patent landscape all the more complicated. And within each gene, variants are highly variable in how common they are and how powerfully they raise cancer risk, making a fair assessment of monetary value even more complex.

### VII.E. Establish a Framework for Cross-Licensing or Patent Pooling

There are precedents for cross-licensing and patent pools to allocate royalties and rewards for copyright (ASCAP)^151^ and patents (e.g., MPEG-LA and VIA Licensing Alliance).^152^ The virtue of these options is that they do not require direct action by Congress or the courts but emerge from voluntary decisions of firms and others holding patent rights.

The idea of gene patent pools has loomed in the background for years. MPEG-LA proposed a pool for gene patents in 2010, as the *Mayo* and *Myriad* cases wended their way to the Supreme Court,^153^ and a decade later for CRISPR patents.^154^ Van Overwalle edited an entire volume to address *Gene Patents and Collaborative Licensing Models*.^155^ Esther van Zimmeren’s 650-page PhD dissertation further explored pooling, cross-licensing and other options, part of which was summarized in a 2011 article.^156^ And more recently, Olson and Ducci flagged pooling and cross-licensing as options.^157^

It has proven more difficult to develop pools in biomedicine and life sciences, however, than for information and communication technologies. Pools and cross-licensing agreements require gatekeepers to ensure that patents entering the pool are valid and enforceable, as well as a system for allocating royalties. It also entails a joint agreement to enforce against unlicensed users. Could these be developed for molecular diagnostics? To date, such efforts have not gained traction. Pooling and cross-licensing nonetheless remain possibilities, although they clearly present a major challenge.

### VII.F. Create Statutory Exemptions from Infringement Liability or Develop Case Law Exceptions through Litigation

The Secretary’s Advisory Committee on Genetics Health and Society proposed a statutory exemption for genetic testing for patient care, and for research.^158^ One justification was the need to verify a prior test or obtain a second opinion in clinical care; another was the need for freedom to operate in doing research. A statutory exemption from infringement liability was the Committee’s foremost recommendation. It elicited a dissent from three members.^159^ A press conference sponsored by the Association of University Technology Managers and the Biotechnology Innovation Organization was scheduled the day before the report’s release to undermine the committee’s recommendation.^160^ Consensus, or at least unanimity, proved elusive. Statutory reform to create a research or ‘second opinion or verification’ exemption might well be difficult, and the process contentious.

All these options come with complications, and none would eliminate uncertainties entirely. Many of the options could emerge from case law, even if the Supreme Court decisions were abrogated by statute. Indeed, experts agreed that if §101 patentable subject matter were revised to make ‘everything is patentable,’ per Risch, then case law would move from §101 patent-eligibility to §101 utility, §102 novelty, §103 obviousness (or inventive step), and §112 (enablement, written description, and definiteness/best mode). The ability of other patent doctrines to weed out unduly broad claims was raised with the Court of Appeals in *Myriad*, but then overshadowed by the focus on patent-eligibility.^161^ The courts would wrestle with the facts of situations using different doctrines of patent law to reach desired policy outcomes. There was no consensus on any one option among our experts. Moreover, other innovation incentives such as payment, coverage, and regulatory exclusivity are other legal tools in the toolbox.

Another general option is to consider effects on innovation when making decisions currently framed as health policy. Just as some legal scholars lost sight of how health policies affected innovation, those making health policies neglected how coverage and payment policies can undermine innovation. Patent jurisprudence largely ignored effects on access and public health because patents were conceived solely as innovation policy. The reverse mistake—failing to appreciate how coding, coverage, and reimbursement affect innovation—is equally problematic. Yet, payment and coverage have flexibilities that patents lack. They are much more readily tailored to individual tests and indications and more directly connected to revenue streams. If the purpose of policy change is to encourage molecular diagnostic innovation, then coverage and payment seem better places to start than patent-eligibility.

## VIII. SUMMARY

Current jurisprudence surrounding patentable subject matter breeds uncertainty regarding claims on both methods and compositions of matter. Restoring patent-eligibility by statutory definition in §101 would reduce some uncertainty over patentable subject matter, but might simply shift litigation—and uncertainty while case law accumulated—to challenges under other patent law doctrines. That is, restoring patent-eligibility might reduce uncertainty in one domain of patent law while fomenting uncertainty and inviting patent litigation on other grounds without reducing uncertainty overall.

Efforts to revise §101 are also apt to elicit strong conflict among constituencies, similar to the inability to find common ground regarding the recommendation for an exemption from infringement liability for second opinions and verification testing. Stakeholders in the fight—firms with starkly different business models and patent preferences, the patent bar, and disease advocacy groups—all have armies of advocates and lobbyists at their disposal. A carefully crafted exclusion from patent-eligibility might be possible to craft that would improve the status quo, but the proposed statutory language does not address the core concerns about patents affecting molecular diagnostics that gave rise to *Mayo* and *Myriad* in the first place. Instead, the proposed language in S. 2140 would revert to pre-*Mayo*-*Myriad* standards.

Fervent disagreement about optimal policy will likely translate to political conflict in restoring patent-eligibility, and invite future litigation should the law revert to pre-*Mayo* status. The future thus seems destined to entail continued disagreement, litigation, debate in Congress, and perhaps incremental change on one or more fronts.

In the meantime, the power of molecular diagnostics seems destined to continue to grow, despite financial cross-winds and legal uncertainty, with multi-gene genetic testing, exome testing, and whole-genome analysis becoming more pervasive and integral to management of cancer. The disruption created by *Mayo* and *Myriad* may prove propitious in having created a period during which fear of infringement liability receded and pushed the door wide open for multi-gene testing, and genome-wide analysis. We make no predictions about whether current legal constraints on patentable subject matter will continue or be modified by statue—or how statutory change would affect innovation—but clinical practices entailing multi-gene and whole genome analysis will surely continue to advance apace.

## Footnotes

1 Concert Genetics, A Guide to BRCA Testing for Health Plans, November 2016.

2
United States Patent and Trademark Office, Patent Eligible Subject Matter: Public Views on the Current Jurisprudence in the United States (2022).

3 Patent-Eligibility Restoration Act of 2023, S. 2140, 118th Cong. (2023).

4 GovTrack, *S. 2140: Patent Eligibiliity Restoration Act of 2023*, https://www.govtrack.us/congress/bills/118/s2140 (accessed Oct. 22, 2023).

5
*See* Sergey Nurk et al., *The Complete Sequence of a Human Genome*, 376 Science 44 (2022).

6 Diamond v. Chakrabarty, 447 U.S. 303 (1980).

7 Bilski v. Kappos, 561 U.S. 593 (2010).

8 Mayo Collaborative Servs. v. Prometheus Labs., Inc., 566 U.S. 66 (2012).

9 Ass’n for Molecular Pathology v. Myriad Genetics, Inc., 569 U.S. 576 (2013).

10 As indicators of the intense feeling among patent practitioners, Gene Quinn of IP Watchdog labeled it a ‘frivolous lawsuit’ and doubled down after the District Court decision, contending it was not ‘a big deal’ and would be short-lived. *See* Gene Quinn, *ACLU Files Frivolous Lawsuit Challenging Patents*, Patents & Intellectual Property Law (May 14, 2009), https://ipwatchdog.com/2009/05/14/aclu-files-frivolous-lawsuit-challenging-patents/id=3417/; Gene Quinn, *Hakuna Matada, the ACLU Gene Patent Victory Will Be Short Lived*, Patents & Intellectual Property Law (Mar. 31, 2010), https://ipwatchdog.com/2010/03/31/hakuna-matada-the-aclu-gene-patent-victory-will-be-short-lived/id=9925/. Dale Halling urged that the American Civil Liberties Union be sanctioned, arguing ‘the ACLU either purposely deceived the court or is guilty of gross negligence about the facts plead in the case.’ *See* Dale B Halling, *ACLU Should Be Hit With Rule 11 Sanctions, Patents & Intellectual Property Law* (Nov. 20, 2009), https://ipwatchdog.com/2009/11/20/aclu-should-be-hit-with-rule-11-sanctions/id=7351/. Eric Guttag echoed the call for sanctions on ACLU for ‘gross and deliberate misrepresentation of what Myriad’s patents cover.’ *See* Eric Guttag, *Foaming at the Mouth: The Inane Ruling in the Gene Patents Case, Patents & Intellectual Property Law* (Mar. 30, 2010), https://ipwatchdog.com/2010/03/30/foaming-at-the-mouth-the-inane-ruling-in-the-gene-patents-case/id=9911/. The 9-0 unanimous decision of the Supreme Court in which ACLU largely prevailed suggests the Justices may not agree this case was frivolous or warranted sanctions against ACLU.
*For a summary of the public significance*, *see*  Jorge L. Contreras, Genome Defense: Inside the Epic Legal Battle to Determine Who Owns Your DNA (Chapel Hill, NC: Algonquin, 2021), and Jacob S. Sherkow, Robert Cook-Deegan and Henry T. Greely, *The Myriad Decision at 10,* 25 Ann. Rev. Hum. Genet. Genomics (2024) https://doi.org/10.1146/annurev-genom-010323-011239.

11 Alice Corp. Pty. Ltd v. CLS Bank Int’l, 573 U.S. 208 (2014).

12 This “conventional wisdom” is taken from a Chat GPT4 response to ‘explain how Mayo v Prometheus and Association for Molecular Pathology v Myriad changed genetic testing’, May 21, 2023, available at: https://osf.io/p8vte.

13 Quote taken from the introduction to an interview with Ellen Matloff, *Gene Patent Decision 10 Years: State of Genetic Testing*, Mendelspod (Jun. 15, 2023), https://mendelspod.com/podcasts/gene-patent-decision-10-years-state-genetic-testing-ellen-matloff-my-gene-counsel.

14
*See* Jacob S. Sherkow, Robert Cook-Deegan and Henry T. Greely, *The Myriad Decision at 10,* 25 Annu. Rev. Hum. Genet. Genomics, https://doi.org/10.1146/annurev-genom-010323-011239. *See also* Emanuela Gambini, *In the Aftermath of the ‘Myriad Case’*—*Myriad is Denied Preliminary Injunction against Ambry Genetics*, 5 Eur. J. Risk Regul. 407, 412 (2014); and Andrew Pollack, *Myriad Genetics Ending Patent Dispute on Breast Cancer Risk Testing*, New York Times (Jan. 27, 2015). *Summarized also in*  Jorge L. Contreras, The Genome Defense: inside the epic battle to determine who owns your dna at 337 (2021).

15 Ariosa Diagnostics, Inc. v. Sequenom, Inc.*,* 788 F.3d 1371 (Fed. Cir. 2015).

16
*See generally* Donald Zuhn, *Top Five Stories of 2016*, PatentDocs (Jan. 8, 2017), https://www.patentdocs.org/2017/01/top-five-stories-of-2016.html.

17 Roche Molecular Systems, Inc. v. Cepheid, 905 F.3d 1363 (Fed. Cir. 2018).

18 Cleveland Clinic Foundation v. True Health Diagnostics LLC, 760 Fed. Appx. 1013 (Fed. Cir. 2019).

19 Athena Diagnostics, Inc. v. Mayo Collaborative Services., Llc, 927 F.3d 1333 (Fed. Cir. 2019) (Hughes, J., concurring).

20 Ryan Davis, *Kappos Calls for Abolition of Section 101 of Patent Act*, Law 360 (Apr. 12, 2016), https://www.law360.com/articles/783604/kappos-calls-for-abolition-of-section-101-of-patent-act.

21 Jorge A. Goldstein, Michelle K. Holoubek & Krishan Y. Thakker, *The Time Has Come to Amend 35 U.S.C. § 101*, 44 Aipla Q. J. 171 (2016).

22 Judge Paul R. Michel, *Testimony Before the Subcomm. on Intellectual Propert*y of the *S. Comm. on the Judiciary*, 116th Congress (2019), available at https://www.judiciary.senate.gov/imo/media/doc/Michel%20Testimony.pdf.

23 Athena Diagnostics, *supra* note 19, at 9.

24
Concert Genetics, Current Landscape of Genetic Testing (2018), available at https://www.concertgenetics.com/resources/2018-current-landscape-genetic-testing/. The number of tests subsequently increased to 175,000; See https://www.concert.co/about/ under“2012”time-line.

25
*Id.* at 10.

26
*See*  Donna P. Suchy, Supplemental Comments Related to Patent Subject Matter Eligibility, American Bar Association (2017).

27
*See*  American Intellectual Property Law Association, Aipla Legislative Proposal and Report on Patent Eligible Subject Matter (March 2017).

28
*See*  Intellectual Property Owners Association, Proposed Amendments to Patent Eligible Subject Matter Under 35 U.S.C*.* § 101 (2017), followed by a May 2018 joint proposal from AIPLA and IPO available at https://www.aipla.org/advocacy/legislative/joint-aipla-ipo-proposal-on-patent-eligibility.

29 Thom Tillis, *Draft Outline of Section 101 Reform*, Thom Tillis, U.S. Senator for North Carolina, available at: http://www.tillis.senate.gov/services/files/3491a23f-09c3-4f4a-9a93-71292704c5b1.

30 S. 2140, *supra* note 3.

31 Patent-Eligibility Restoration Act of 2022, S. 4734, 117th Cong. (2022).

32
*See* Isabelle Huys et al., *Gene and Genetic Diagnostic Method Patent Claims: A Comparison Under Current European and US Patent Law*, 19 Eur. J. Hum. Genet. 1104 (2011).

33 The claims relevant to genetic testing include claims to DNA fragments whose sequence is found in *BRCA1* or *BRCA2* genes. Such claims are exemplified by claims 5 and 6 of U.S. patent 5747282 (′282): ‘the isolated DNA having at least 15 nucleotides of the sequence…’ of claims 1 and 2 (claim 2 being the cDNA sequence of *BRCA1,* and claim 1 broadening to include variants in the DNA sequence that would produce the same BRCA1 protein). As Kepler, et al., showed, claim 5 is highly metastatic, identifying over one million sequences in the reference human genome. *See* Thomas Kepler et al., *Metastasizing patent claims on BRCA1*, 95 Genomics 312 (2010). Eighty per cent of the human genes in GenBank in 2010 included sequences that would be covered by this claim; sequencing any one of those genes would entail making molecules that appear to be claimed in the ‘282’, and thus infringe. Why? Because these claims cover DNA encoding any protein with five amino acids in the same order as any stretch of five amino acids in the BRCA1 protein. James Martinell, a senior USPTO examiner flagged this problem in rejecting NIH’s 1991 (later abandoned) patent applications on Expressed Sequence Tags. See James Martinell, U.S. Patent Application No. 07/837195 (filed Feb. 12, 1992) (titled ‘Sequence Characteristics of Human Genome Transcription Product’ by J. Craig Venter et al.), *reprinted in part in* 11 Biotechnology L. Rep. 578, 578–96 (1992). However, Martinell’s precedent does not appear to have carried into examination of the ‘282 patent 5 years later by a different examiner. These claims may speak to errant prosecution and examination, as claims 5 and 6 were scored as ‘subordinate’ because they referred to claims 1 and 2, yet they expand rather than narrow the scope of the patent. Such claims might well be judged invalid if challenged, but these and similar claims in many other gene patents were granted and presumed valid until the *Myriad* case. They thus implied infringement liability for any diagnostic test that entailed sequencing fragments of DNA that included short *BRCA1* sequences. This would include targeted sequencing, amplification of DNA containing fragments with the claimed sequences, sequencing all expressed regions (exome sequencing), or whole-genome analysis.

34 Ass’n for Molecular Pathology v. U.S. Patent & Trademark Office, 653 F.3d 1329 (Fed. Cir. Jul. 29, 2011; and again Aug. 16, 2012).

35 Judge Sweet’s position is close to that adopted in the subsequent case heard by the High Court of Australia in *D’Arcy v Myriad*, which invalidated the core claim to isolated *BRCA1* DNA, and reversed two lower court rulings that had upheld Myriad’s claim.

36 Brief for the United States as Amicus Curiae in Support of Neither Party, Ass’n for Molecular Pathology v. Myriad Genetics, Inc., No. 12-398 (2013).

37 Brief for Amicus Curiae Eric S. Lander in Support of Neither Party, Ass’n for Molecular Pathology v. Myriad Genetics, Inc., No. 12-398 (2013); addressing cDNA at pp. 26–29, and naturally occurring gene fragments at pp. 6–18, esp. footnote 25.

38 The Supreme Court’s incoherence on the physical–chemical versus informational role of DNA is illustrated by three quotes from its decision. On page 15, the Court cites DNA’s information content: ‘Myriad’s claims are simply not expressed in terms of chemical composition… Instead, the claims understandably focus on the genetic information encoded in the *BRCA1* and *BRCA2* genes.… [I]ts claim is concerned primarily with the information contained in the genetic sequence, not with the specific chemical composition of a particular molecule.*’* [emphasis added; 569 US 576 at 15] On the next page, it shifts to the chemical argument: ‘…[C]reation of a cDNA sequence from mRNA results in an exons-only molecule that is not naturally occurring… the lab technician unquestionably creates something new when cDNA is made’ [emphasis added; at 16]. And, in its final sentence, it conflates both rationales: ‘We merely hold that genes and the information they encode are not patent eligible under §101 simply because they have been isolated from the surrounding genetic material’ [emphasis added; at 18]. There was extensive commentary that followed the *Myriad* decision. The late Dan Burk looked for clues connecting *Mayo* and *Myriad*. *See* Dan L. Burk, *The Curious Incident of the Supreme Court in Myriad Genetics,* 90 Notre Dame L. Rev. 505 (2014), and *See* Jacob Sherkow, et al., Ethical, legal, and social issues in the Earth BioGenome Project, 119 Pnas (2022).

39 The High Court of Australia did not follow the Thomas doctrine. In a 3–0 unanimous ruling, it used the information rationale to invalidate Myriad’s challenged composition of matter claim, noting DNA’s ‘substance is information embodied in arrangements of nucleotides. The information is not “made” by human action. It is discerned… [a DNA sequence] is a characteristic of the human being from whom the nucleic acid is isolated, a characteristic not shared by all human beings. It has nothing to do with the person who isolates the nucleic acid bearing the mutant sequence’ See D’Arcy v. Myriad Genetics Inc, (High Ct. Australia 2015) at 4.

40 Knowles and Prosser note: ‘Examples of marketed drugs that have been discovered in nature and then isolated and used in a non-naturally occurring form with important therapeutic uses include penicillin, tetracycline, [erythropoietin], adriamycin, insulin, vincristine, vinblastine, streptomycin, and Vitamin B12’ [generic name erythropoietin substituted for trade name Epogen® that was used in the original]. *See* Sherry Knowles & Anthony Prosser, *Unconstitutional Application of 35 U.S.C. § 101 by the U.S. Supreme Court*, 18 J. Marshall Rev. Intell. Prop. L. 144, 146 (2018). They could have added thyroxine and corticosteroids, which had their own curious patent histories from mid-20^th^ Century discovery, and other protein therapeutics developed by ‘gene jockeys’ that were first generation products of modern biotechnology. *See*  Graham Dutfield, That High Design of Purest Gold: A Critical History of the Pharmaceutical Industry 1880–2020 at 217–274 (2020); Nicholas Rasmussen, *Of ‘Small Men,’ Big Science and Bigger Business: The Second World War and Biomedical Research in the United States*, 40 Minerva 115 (2002); Nicholas Rasmussen, Gene Jockeys: Life Science and the Rise of Biotech Enterprise (2014).

41
*See* Michael Risch, *Everything is Patentable*, 75 Tenn. L. Rev. 591 (2008).

42 Myriad’s price for assembling the sequence of *BRCA1* and *BRCA2* was initially $2400 in 1996–1998 and rose to $3400 by 2013; in 2006, Myriad added another separate $700 test for large structural changes in the genes.

43 Bryson Opinion concurring in part and dissenting in part in Ass’n for Molecular Pathology, et al., v. U.S. Patent and Trademark Office, et al., 653 F.3d 1329 (Fed. Cir. 2011) at 8–9, 14, and esp. 16.

44
*See* Michael A. Heller & Rebecca S. Eisenberg, *Can Patents Deter Innovation? The Anticommons in Biomedical Research*, 280 Science 698 (1998).

45
*See* Robert Cook-Deegan et al., *Impact of Gene Patents and Licensing Practices on Access to Genetic Testing for Inherited Susceptibility to Cancer: Comparing Breast and Ovarian Cancers with Colon Cancers*, 12 Genet. Med. S15 (2010).

46
*See* Robert Cook-Deegan & Christopher Heaney, *Gene patents and licensing: Case studies prepared for the Secretary’s Advisory Committee on Genetics, Health, and Society*, 12 Genet. Med. S1-S2 (2010).

47
*See* James Evans, *Putting patients before patents*, 12 Genet. Med. S3-S4 (2010).

48
Secretary’s Advisory Committee on Genetics, Health, and Society, Patents and Licensing Practices and Their Impact on Patient Access to Genetic Tests at 4 (2010), available at https://repository.library.georgetown.edu/handle/10822/515456.

49
*See* Julia Carbone & E. Richard Gold, *Myriad Genetics: In the Eye of the Policy Storm*, 12 Genet. Med. S39 (2010).

50
*See*  Jorge Contreras, The Genome Defense: Inside the Epic Battle to Determine Who Owns Your DNA at 324–325 (2021).

51
*See* Cook-Deegan, et al., *supra* note 46.

52 In May 2022 we received a personal communication from Concert Genetics that detailed the total number of genetic tests for hereditary cancer, what category the test was, whether it was a panel or single-gene, what lab offered the test, the name of the test, and what gene targets were included. From these data, we calculated the number of companies offering tests for *BRCA1/2*. The spreadsheets listing the patents, genes, claims, and other data are on deposit at Open Science Framework at https://osf.io/37x2r/ (DOI 10.17605/OSF.IO/37X2R). Which genes were tested by which companies is also detailed in Janis Geary, Mary Majumder, Christi Guerrini & Robert Cook-Deegan 8(4) Jama Oncology 637 (2022), with underlying data available at https://osf.io/qwyg2/.

53 Myriad’s MyRisk® test was initially 25 genes, then 28, and now (mid-2024) 48 genes associated with cancer of breast, ovary, uterus, colon and rectum, skin, pancreas, stomach, prostate, kidney, lung, endocrine glands, and others. *See Hereditary Cancer 48 Gene Panel Associated Syndromes*, MyRisk (May 19, 2022), https://myriad-web.s3.amazonaws.com/myRisk/GeneTable_Syndromes_0422.pdf.

54 Judge Shelby’s 2014 decision memorandum also recounts part of this chronology. *See* Memorandum Decision and order denying plaintiffs motion for preliminary injunctions, University of Utah Research Foundation v. Ambry Genetics, Case 2:13-cv-00640-RJS Document 185, US District Court for Utah, 2014.

55
*See* Patricia A. Deverka et al., *Payer Reimbursement Practices and Incentives for Improving Interpretation of Germline Genetic Testing*, 10 J. l. Biosci. (2023).

56 SACGHS. Coverage and Reimbursement of Genetic Tests and Services: Report of the Secretary’s Advisory Committee on Genetics, Health, and Society (2006). Available at http://hdl.handle.net/10822/711781.

57
*See* Cook-Deegan, et al., *supra* note 46.

58
*Ibid.*

59
*Id.*

60
*See* Deverka et al., *supra* note 55.

61 Details about the complex interactions among coding, reimbursement, and coverage are reviewed in this special issue by Deverka et al., *supra* note 55.

62
*See* Deverka et al., *supra* note 55.

63 Reference to CPT codes is done with permission from AMA.

64
*See* Deverka et al., *supra* note 55.

65 U.S. Department of Health & Human Services, Office of the Inspector General, *Trends in Genetic Tests Provided under Medicare Part B Indicate Areas of Possible Concern*, HHS OIG Data Brief A-09-20-03027, December 2021.

66 Protecting Access to Medicare Act of 2014, Pub. L. No. 113-93, 128 Stat. 1053 (codified at 42 U.S.C. § 1395 m-1) (2014).

67
*See* Rachel Sachs, *The Accidental Innovation Policymakers*, 72 Duke l. j. 1431 (2023).

68
*See* Lisa Larrimore Ouellette, *Patentable Subject Matter and Nonpatent Innovation Incentives*, 5 Uc Irvine L. Rev. 1115 (2015).

69 Medical Device Amendments of 1976, Pub. L. No. 94-295, 90 Stat. 539 (1976).

70 See appendix to Office of Technology Assessment, u.s. Congress, Human Gene Therapy- a Background Paper ota-bp-ba-32 (1984).

71 See generally Neil A. Holtzman, Proceed with Caution: predicting genetic risks in the recombinant dna era (1989).

72
*See* Neil A Holtzman, *The FDA and the Regulation of Genetic Tests*, 41 Jurimetrics 53 (2000).

73
Sally Howard, Department of Health human Services - Food and Drug Administration (2014), available at https://www.fda.gov/media/89316/download.

74 Gail Javitt, *FDA Regulation Of Laboratory Developed Tests: A Long Saga*, Law 360 (Dec. 15, 2016), https://www.law360.com/articles/873238/fda-regulation-of-laboratory-developed-tests-a-long-saga.

75 21st Century Cures Act of 2016, Pub. L. No. 114-255 (2016).

76
Food and Drug Administration (FDA), Discussion Paper on Laboratory Developed Tests (2017), available at https://www.fda.gov/media/102367/download.

77 Payment Choice Act, H.R. 4128, 117th Congress.; VALID Act of 2021, S. 2209, 117th Congress (2021).

78
*See* Rachel Sachs, *FDA User Fee Reauthorization: Contextualizing The VALID Act*, Healthaffairs, Jun. 23, 2022, https://www.healthaffairs.org/do/10.1377/forefront.20220622.414686.

79
*See* Mark B McClellan & Scott Gottlieb, *Congress Needs to Update FDA’s Ability to Regulate Diagnostic Tests,*  Stat, Dec. 5, 2022, https://www.statnews.com/2022/12/05/congress-update-fda-regulations-diagnostic-tests-cosmetics.

80 VITAL Act of 2021, S. 1666, 117th Congress (2021).

81 Proposed Rule Medical Devices: Laboratory Developed Tests (codified at 21 D.F.R. pt. 808; RIN 0910-Al85). Available at https://www.reginfo.gov/public/do/eAgendaViewRule?pubId=202304&RIN=0910-A.

82 Department of Health and Human Services, Food and Drug Administration, *Medical Devices; Laboratory Developed Tests* 88 FR 68006; Docket No. FDA-2023-N-2177, available at: https://www.federalregister.gov/documents/2023/10/03/2023-21662/medical-devices-laboratory-developed-tests. The announcement, final rule, FAQs and related documents are available at https://www.fda.gov/medical-devices/in-vitro-diagnostics/laboratory-developed-tests.

83
*U.S. FDA Plans Laboratory-Developed Test Rulemaking and Launches Pilot Program*, Sidley (July 5, 2023), available at: https://www.sidley.com/en/insights/newsupdates/2023/07/us-fda-plans-laboratory-developed-test-rulemaking-and-launches-pilot-program.

84
*See* Jeffrey Gibbs et al., *FDA’s Summer Plans May Include LDT Rulemaking*, FDA Law Blog (June 20, 2023), available at: https://www.thefdalawblog.com/2023/06/fdas-summer-plans-may-include-ldt-rulemaking. See also https://www.fda.gov/medical-devices/in-vitro-diagnostics/laboratory-developed-tests (accessed 3 May 2024).

85
*See* Rebecca S. Eisenberg, *The Role of the FDA in Innovation Policy*, 13 Mich. Telecomm. & Tech. l. Rev. 345 (2007).

86
*See* Michael M. Hopkins & Stuart Hogarth, *Biomarker Patents for Diagnostics: Problem or Solution?* 30 Nat. Biotechnol. 498 (2012).

87
*See*  Andrew Sagar et al., Patent-Eligibility and Precision Medicine: Emerging Technologies, Legal Flux and Empirical Methods, Patenting Biotechnical Innovation (Elgar Publishing) 19 (2022).

88
*See* Kathryn Maxson Jones, Rachel Ankeny & Robert Cook-Deegan, *The Bermuda Triangle: The Pragmatics, Policies, and Principles for Data Sharing in the History of the Human Genome Project*, 41 J. Hist. Biol. 693 (2018).

89
*See*  Georgina Ferry & John Sulston, The Common Thread: a Story of Science, Politics, Ethics, and the Human Genome at 139–147 (2002).

90 Myriad published rarely and did not include details sufficient for verification of its conclusions when it did publish. See Robert Cook-Deegan et al., *The Next Controversy in Genetic Testing: Clinical Data as Trade Secrets?* 21 Eur j. Hum. Genet. 585 (2013). Conley, et al., also reviewed this history. See Conley, et al., *Myriad after Myriad: The Proprietary Data Dilemma*, 15(4) N. Carolina J. Law Tech. 597 (2014). Myriad’s first medical director, Thomas Frank, was an avid data-sharer, and helped establish the open science Breast Cancer Information Core that was established and maintained by Larry Brody and colleagues at the National Human Genome Research Institute. Myriad’s public data-sharing changed after Frank’s death when Myriad’s CEO, Peter Meldrum, became aware of the data-sharing practices. That policy recently changed. Under Myriad’s new CEO, Paul J. Diaz, Myriad announced in November 2022 that it would start sharing variant data with the public database ClinVar. *See* Ellen Matloff, *Myriad Genetics (MYGN) To Share Guarded BRCA Variant Information In Public Database*, Forbes (Nov. 3, 2022), https://www.forbes.com/sites/ellenmatloff/2022/11/03/myriad-genetics-mygn-to-share-guarded-brca-variant-information-in-public-database/?sh=73a55761a772. Myriad piloted a ClinVar submission of over 40 variants in March 2023, and contributed data on 2581 variants in July 2023, the first of regular quarterly deposits. *See* Personal communication with Karla Bowles, Myriad Genetics (May and August 2023).

91 Judge Robert Sweet commented on this perverse effect of the patents in his 2010 district court decision destined for the Supreme Court. Judge Robert Shelby called it out even more forcefully in his 2014 decision about the follow-on lawsuits after *Myriad*, noting: ‘…[T]he practical result of Myriad’s patents has been to hinder or halt follow-up research, data-sharing, patient testing, and the creation of additional and more affordable technologies for *BRCA1* and *BRCA2* testing… Myriad has declined to publicly share critical information regarding its classification of variants, including with its own patients. Instead, Myriad retains that information in a private database. In so doing, Myriad distorts rather than serves the patent system’s goal of public disclosure in exchange for exclusive rights. In this way, Myriad has chosen a commercial path that turns much of our patent system policy on its head.’ *See* Memorandum Decision, *supra* note 54.

92
*See* J.M. Eggington et al., *A comprehensive laboratory-based program for classification of variants of uncertain significance in hereditary cancer genes*, 86 Clin. Genet. 229 (2013).

93
*See* Robert Cook-Deegan, et al., *The Next Controversy in Genetic Testing: Clinical Data as Trade Secrets?*, 21 Eur j. Hum. Genet. 585 (2013).

94 Simon and Sichelman addressed this anomaly, in which ‘Data-Generating Patents’ can become the basis for trade secrets. See Brenda M. Simon & Ted Sichelman, *Data-Generating Patents*, 111 Nw. u. l. Rev. 377 (2017). Trade secrets are often framed as a trade-off with patents—that secrecy would replace public disclosure without the open disclosure of patents. Yet some patents create exclusive rights to collect information, and can become the source of trade secrets that do not expire. Simon and Sichelman note that the adverse effects of such patents could be addressed by research exemptions from infringement liability, independent invention exceptions, patent misuse doctrines, or mechanisms to force disclosure. As a matter of patent policy, as Judge Shelby observed (footnote 91 *supra*), the trade secret database of cancer-related variants was attributable to the patents. That is, patent exclusivity bred trade secret exclusivity rather than being a trade-off of open disclosure in the patent as a trade-off for temporary exclusive rights. The broad claims granted to Myriad initially covered any way of detecting variants and any DNA molecular of longer than 15 base pairs corresponding to *BRCA1* or *BRCA2*, and this was conventional patent practice at the time; but the patents did not describe the hard work of identifying which variants had clinical significance (beyond those specified in the patent). This is immensely significant, because of the over 70,000 variants in BRCA1/2, only about 7 per cent are understood to confer high risk of cancer. See BRCA Exchange, BRCAexchange.org. At Myriad, the transient exclusive patent right directly gave rise to permanent exclusive control of data because no other US labs did testing at scale to find new variants. Myriad dramatically improved its ability to interpret genetic test results, but the knowledge was not shared. The proprietary data became a proprietary asset.

95 National Research Council, National Academy of Sciences, *Toward Precision Medicine: Building a Knowledge Network for Biomedical Research and a New Taxonomy of Disease* (2011).

96 Global Alliance for Genomics and Health (GA4GH), *Creating a Global Alliance to Enable Responsible Sharing of Genomic and Clinical Data*, June 3, 2013, https://www.ga4gh.org/document/white-paper/ (accessed Mar. 9, 2024).

97 The inaugural white paper was released on June 3, 2013, just 10 days before the Supreme Court decision in *Myriad* was handed down.

98
*See* Melissa S. Cline et al., *BRCA Challenge: BRCA exchange as a global resource for variants in BRCA1 and BRCA2*, 14 Plos Genet. (2018).

99
*See* Christophe Béroud et al., *BRCA Share: a collection of clinical BRCA gene variants.* 37 Hum. Mutat. 1318–28 (2016).

100
*See* Cline, *supra* note 98.

101 Personal Communication with Melissa Cline, BRCA Exchange (21 June 2022).

102 BRCA Exchange, BRCAexchange.org (accessed Mar. 9, 2024).

103 Indeed, several firms argued *against* gene patents during the *Myriad* case, including GeneDx, Invitae, and Ambry, in no small part because of how patents bred trade secrecy. (*See* amicus briefs from Association for Molecular Pathology, GeneDx, and Invitae at SCOTUSblog: https://www.scotusblog.com/case-files/cases/association-for-molecular-pathology-v-myriad-genetics-inc/). ‘Industry’ was thus divided, not universally in support of diagnostic gene patents. And indeed, on the tenth anniversary of the *Myriad* decision, Myriad’s new CEO, Paul Diaz, stated that genes should not be patented and that the Supreme Court decisions did not hinder innovation, and Myriad posted a position statement on Twitter at https://twitter.com/myriadgenetics/status/1668606258578857984/photo/1 eschewing gene patents and announcing the data-sharing policy for ClinVar. *See* Turna Ray, *Decade After SCOTUS Gene Patents Ruling, Precision Medicine and Test Innovation Impact Under Debate*, GenomeWeb (Jun. 13, 2023), https://www.genomeweb.com/molecular-diagnostics/decade-after-scotus-gene-patents-ruling-precision-medicine-and-test (accessed Mar. 9, 2024).

104
*See* Melissa J. Landrum et al., *ClinVar: Improving Access to Variant Interpretations and Supporting Evidence*, 46 Nucleic Acids Res. D1062 (2018).

105
*See* Steven M. Harrison et al., *Clinical Laboratories Collaborate to Resolve Differences in Variant Interpretations Submitted to Clinvar*, 19 Genet. Med. 1096–1104 (2017).

106
*See* Steven M. Harrison et al., *Scaling Resolution of Variant Classification Differences in ClinVar Between 41 Clinical Laboratories Through an Outlier Approach*, 39 Hum. Mutat. 1641 (2018).

107
*See* Juli Bollinger et al., *BRCA1/2 Variant Data-Sharing Practices*, 47 J. L. MED. ETHICS 88 (2019). Also worth noting, while most laboratories did not mention it, they also all used the open source human reference resources managed by the Genome Reference Consortium, usually Build 37 (GRCh37; 2009 https://www.ncbi.nlm.nih.gov/datasets/genome/GCF_000001405.13/) or Build 38 (GRCh38 or the improved GH38; 2019 https://gatk.broadinstitute.org/hc/en-us/articles/360035890951-Human-genome-reference-builds-GRCh38-or-hg38-b37-hg19). Those linear reference sequences are used in the first step of clinical genome sequencing, by mapping the raw sequence reads to the reference sequence before identifying variants.

108 Ludmila Leiva, *Elizabeth Holmes Theranos Scandal: A Complete Timeline,* Refinery 29 (Jan. 29, 2019), https://www.refinery29.com/en-us/2019/01/222855/theranos-scandal-timeline-what-happened-elizabeth-holmes-documentary (accessed Mar. 9, 2024).

109
*See generally*  John Carreyrou, Bad Blood: secrets and lies in a silicon valley startup (2020).

110 Sebastian Mallaby, *How Theranos Fooled V.C.s*, New York Times (Jan. 24, 2022), https://www.nytimes.com/2022/01/24/opinion/theranos-venture-capital.html (accessed March 9, 2024).

111 Bobby Allyn, *Elizabeth Holmes has Started Her 11-Year Prison Sentence. Here’s What to Know*, NPR (May 30, 2023), https://www.npr.org/2023/05/30/1178728092/elizabeth-holmes-prison-sentence-theranos-fraud-silicon-valley (accessed Mar. 9, 2024).

112 Sarah Jackson, *Meet Sunny Balwani: Theranos No. 2 and Elizabeth Holmes’ Ex-Boyfriend Who’s Serving a 13-Year Prison Sentence and Was Just Ordered to Pay $452 Million in Restitution With her*, Business Insider (Jun. 2, 2023), https://www.businessinsider.com/who-is-ramesh-sunny-balwani-elizabeth-holmes-ex-theranos-trial-2022-3 (accessed Mar. 9. 2024).

113 Steve Brozak, *Theranos Debacle May Scare Investors Away from Diagnostic Technology*, Stat News (Apr. 13, 2016), https://www.statnews.com/2016/04/13/theranos-debacle-threatens-investment-diagnostic-testing (accessed Mar. 9, 2024).

114
*See* David O Taylor, *Patent-Eligibility and Investment*, 41 Cardozo l. Rev. 2019 (2019).

115 David S. Olson & Fabrizio Ducci, *Patenting Genetic Information*, 98(4) Indiana l. j. 1180 (2023).

116
*See* Sasha Hoyt, *The Impact of Uncertainty Regarding Patent Eligible Subject Matter for Investment in U.S. Medical Diagnostic Technologies*, 79 Wash. Lee l. Rev. 397 (2022).

117 Hoyt went on to infer that ‘the data support[s] the recent calls to Congress for reform of §101.’ *See Id*. Hoyt’s difference-in-difference analysis that found a shortfall in VC funding of medical diagnostics is quite credible. Difference-in-difference analyses are powerful tools for inferring causality, but only if the ‘treatment’ is the only variable distinguishing ‘treated’ from ‘untreated.’ Coverage, reimbursement, regulation, and the Theranos effect confound the interpretation and undermine a simple cause and effect attribution. Using VC investment as the sole metric is also problematic. Most labs that began or expanded genetic testing after *Myriad* already existed and were past VC investment stage or were part of major clinical institutions (clinics, hospitals, or universities) that did not draw on VC. Large firms, including LabCorp and Quest, began BRCA testing post-*Myriad* without resort to VC. Arguing for a deficit in genetic testing innovation confronts the fact that over 74,000 genetic tests were available by 2018, twice the 37,000 available in 2014 after *Alice* reinforced the *Mayo* decision. From 2015 to 2022, the number further escalated from 66,000 to 175,000. *See* Ray, *supra* note 103.

118
*See* Paul R. Michel, *The Supreme Court is Partly to Blame for the Covid-19 Test Kit Shortage*, Stat News (Feb. 7, 2022), https://www.statnews.com/2022/02/07/supreme-court-partly-to-blame-covid-19-test-kit-shortage/; *See also* Joe Patrice, *No COVID Tests at Your Drug Store? Blame the Supreme Court*, Above the Law (Feb. 7, 2022), https://abovethelaw.com/2022/02/no-covid-tests-at-your-drug-store-blame-the-supreme-court/.

119 Judge Michel’s assertion that lack of patent-eligibility accounted for a shortage of Covid tests stands in contrast to the Government Accountability Office finding that FDA received over 3000 requests for emergency use authorization of Covid tests from February 2020 to December 2021, of which 420 were granted. See Government Accountability Office, Fda Took Steps to Help Make Tests Available: Policy For Future Public Health Emergencies Needed (2022), available at https://www.gao.gov/assets/gao-22-104266.pdf.

120 Paul Michel et al., *Presenting the Evidence for Patent-Eligibility Reform: Part I*, IPWatchdog (Oct. 6, 2022), https://ipwatchdog.com/2022/10/06/presenting-evidence-patent-eligibility-reform-part-consensus-patent-law-experts/id=151886/.

121 Paul Michel et al., *Presenting the Evidence for Patent-Eligibility Reform: Part II*, IPWatchdog (Oct. 11, 2022), https://ipwatchdog.com/2022/10/11/presenting-evidence-patent-eligibility-reform-part-ii-harm-rd-investment-innovation-u-s-interests/id=151960/.

122 Paul Michel et al., *Presenting the Evidence for Patent-Eligibility Reform: Part III*, IPWatchdog (Oct. 18, 2022), https://ipwatchdog.com/2022/10/18/presenting-evidence-patent-eligibility-reform-part-iii-case-studies-litigation-data-highlight-additional-evidence-harm/id=152193/.

123 Paul Michel et al., *Presenting the Evidence for Patent-Eligibility Reform: Part IV*, IPWatchdog (Oct. 26, 2022), https://ipwatchdog.com/2022/10/26/presenting-evidence-patent-eligibility-reform-part-iv-uncertainty-burdening-litigants-courts-threatening-u-s-competitiveness-national-security/id=152341/.

124 Arti Kaur Rai, Colleen V. Chien & Jenna Clark, Molecular Diagnostics Patenting After Mayo c. Prometheus: An Empirical Analysis, Duke Law School Public Law & Theory Series No. 2023-69 (Nov. 29, 2023), available at: http://dx.doi.org/10.2139/ssrn.4648623.

125 Concert Genetics, *supra* note 24*.*

126
*See* Lori Pressman et al., *The Licensing of DNA Patents by US Academic Institutions: An Empirical Survey*, 24 Nat. Biotechnol. 38 (2006).

127 Bayh-Dole Act P.L. 96–517, 94 Stat. 3015; 35 U.S.C. §§ 200–212.

128 Stevenson-Wydler Act, P.L. 96–480, 94 Stat. 2311; 15 U.S.C. §§ 3701–3710; amended by P.L. 117–286.

129 This is significant because patents and their claims are public information, but licensing data are not public and her study could not examine academic institution licensing agreements. Pressman’s insight could guide future empirical scholarship, but would require access to licensing data. As noted in text, patents licensed for diagnostic use that had some exclusivity, intriguingly, were faster to generate revenues than without exclusivity, signaling a possible investment incentive accelerating speed to market, although the effect was not strong. Reagents were faster to produce revenue than non-DNA diagnostics, in turn faster to return revenues than nucleic-acid based diagnostics.

130
*See* Lori Pressman, *DNA Patent Licensing Under Two Policy Frameworks: Implications for Patient Access to Clinical Diagnostic Genomic Tests and Licensing Practice in the Not-for-Profit Sector*, 6 Life Sci. l. Indus. Rep. 18 (2012).

131 Interviews (*N* = 15) were conducted under Protocol Study 00009507, Arizona State University Institutional Review Board; approved 18 February 2019. This work is covered by a Certificate of Confidentiality under the 21st Century Cures Act. *See* P.L. 114–255; 42 USC 201, §2012 and 2013.

132 eBay Inc. v. MercExchange, L.L.C., 547 U.S. 388 (2006).

133 With the kind assistance of of Osmat Jefferson and others at Lens.org (accessed Mar. 9, 2024), we identified patents whose claims explicitly listed one or more of the 27 genes tested by the 7 labs contributing most data to the ClinVar database, plus Myriad Genetics, as of October 2021: Ambry, GeneDx, ARUP, Invitae, Color, LabCorp, Quest, and Myriad. Those genes were: *BRCA1, BRCA2, MSH2, PTEN, STK11, TP53, ATM, BRIP1, CDH1, EPCAM, NBN, CDK4C, DKN2A, POLE, POLD1, MSH6, PMS2, APC, BMPR1A, MUTYH, SMAD4, BARD1, CHEK2, PALB2, RAD51C, RAD51D,* and *MLH1.* The tranche of patents had to be reviewed manually because one gene, *POLE*, flagged unrelated patents (because it is an English word), and to remove other spurious patents. We then started to classify claims on the most commonly tested genes with most patent claims including *BRCA1/2* and beyond: *MSH6, PMS2, APC, BMPR1A, MUTYH, SMAD4, BARD1, CHEK2, RAD51C,* and *RAD51D.* A spreadsheet with the list of patents, and an explanation of the process are deposited at Open Science Framework. See also Janis Geary et al., *Patents Mentioning 27 Hereditary Cancer Genes* (Jul. 26, 2023), https://osf.io/ua9tv.

134 Claim 2 in the ‘282’ patent, for example, claimed the complementary DNA for *BRCA1.* The University of Utah and Myriad characterized the gene and disclosed the cDNA sequence in the patent. Per the Supreme Court decision in *Myriad,* that claim is not invalid (assuming it meets other tests for validity) and could have been enforced until it expired in 2014. The cDNA claim might have been valuable if someone had developed a therapeutic based on the protein encoded by the *BRCA1* gene, analogous to gene patents that underlie therapeutic proteins such as peptide hormones (e.g., insulin and growth hormone) or enzymes. Yet, a full-length cDNA is not generated in genetic testing, so this claim was not relevant to genetic testing.

135
*See* Wen-Wei Liao et al., *A Draft Human Pangenome Reference*, 617 Nature 312 (2023).

136 The text of the claim is: ‘A method for screening germline of a human subject for an alteration of a BRCA1 gene which comprises comparing germline sequence of a BRCA1 gene or BRCA1 RNA from a tissue sample from said subject or a sequence of BRCA1 cDNA made from mRNA from said sample with germline sequences of wild-type BRCA1 gene, wild-type BRCA1 RNA or wild-type BRCA1 cDNA, wherein a difference in the sequence of the BRCA1 gene, BRCA1 RNA or BRCA1 cDNA of the subject from wild-type indicates an alteration in the BRCA1 gene in said subject.’ US Patent 5710001 makes a similar claim for tumor samples (as opposed to germline testing).

137 This patent was subordinate to the Myriad method and composition-of-matter patent claims (now invalid). The Miami patent was granted in 2008, before *Mayo*. At that time, the Miami patent would have required a license from Myriad to use the invention, since Myriad’s claims encompassed those in the Miami patent. Miami would need a license from Myriad to practice its invention, but Myriad or any other US lab would conversely need a license from Miami to test African American women who carried the Miami-patent-specified variants.

138
BRCAexchange.org (accessed Mar. 9, 2024).

139 See Nandana D. Rao & Brian H. Shirts, *Using Species Richness Calculations to Model the Global Profile of Unsampled Pathogenic Variants: Examples from BRCA1 and BRCA2*, 18 Plos One (2023).

140 KSR Int’l Co. v. Teleflex Inc., 550 U.S. 398 (2007). Holman and Cook-Deegan argued that patent doctrines concerning novelty, nonobviousness, written description and enablement could weed most overly broad claims arising in gene patents. *Brief of* amici curiae *Christopher M. Holman and Robert Cook-Deegan in Support of Neither Party* (Fed. Cir 2010–1406, October 28, 2010) https://dukespace.lib.duke.edu/items/f6842b75-02af-4bba-a38f-03892263f69b (accessed Mar. 9, 2024).

141 Amgen Inc. v. Sanofi, 598 U.S. 594 (2023). Lemley and Sherkow called for a somewhat broader range of enforceable ‘genus’ claims than the Court of Appeals granted. *See*  Mark A. Lemley and Jacob S. Sherkow, *The Antibody Patent Paradox* 132(4) Yale L. J. 910 (2023). This case could become relevant to genetic diagnostics in the sense that, as noted above, past ‘gene patents’ claimed all variants in those DNA molecules, and any method of detecting such variants, analogous to ‘genus’ claims on monoclonal antibodies. The doctrines of enablement, written description and definiteness could be applied to DNA claims, narrowing their scope.

142 S. 2140, *supra* note 2.

143
John Crewdson, A Scientific Mystery, a Massive Cover-up, and the Dark Legacy of Robert Gallo (2003).

144 Amgen Inc. v. Sanofi, *supra* note 141.

145 Maureen A. O’Malley, Adam Bostanci & Jane Calvert, *Whole-Genome Patenting*, 6 Nat. Rev. Genet. 503, 506.

146
*See*  Comments of Jeffrey Lefstin, Peter Menell and Allison Schmitt on the Patent Eligibility Restoration Act of 2023, UC San Francisco Research Paper, forthcoming, posted at SSRN: https://papers.ssrn.com/sol3/papers.cfm?abstract_id=4677636 (accessed Mar. 9, 2024).

147 It is worth noting, however, that our interviews addressed only patent-eligibility and molecular diagnostics. Consensus about uncertainty may not hold across all technological domains. Datzov and Rantanen argue that the levels of uncertainty and inconsistency in applying §101 patentable subject matter doctrine are within norms of other doctrines, based on their empirical analysis of court decisions. See Nikola Datzov and Jason Rantanen *Predictable Unpredictability (Work in Progress)* (July 28, 2023), U Iowa Legal Studies Research Paper No. 2024-04, Iowa L. Rev., forthcoming. Available at SSRN: https://ssrn.com/abstract=4380434 (accessed Mar. 9, 2024); *summarized in*  Jason Ratanen  *The Predictability of the Mayo/Alice Framework—A New Empirical Perspective*  Patently-O (Nov. 15, 2023) at https://patentlyo.com/patent/2023/11/predictability-framework-perspective.html (accessed Mar. 9, 2024).

148 Decision Memorandum, *supra* note 54, at 106.

149 Amgen Inc. v. Sanofi, *supra* note 141.

150 US Patent 7384743, *supra* note 137.

151
American Society of Composers, https://www.ascap.com (last visited May 10, 2023).

152 VIA LA*,*  https://www.via-la.com/about/#overview (last visited May 10, 2023).

153 Donald Zuhn, *MPEG LA Announces Formation of Gene Patent Pool,* Patent Docs (Apr. 21, 2010), http://www.patentdocs.org/2010/04/mpeg-la-announces-gene-patent-licensing-initiative.html.

154 VIA LA, *CRISPR*, http://www.mpegla.com/crispr/initiative/ (last visited May 10, 2023).

155
Geertrui van Overwalle, Gene Patents an Collaborative Licensing Models: patent pools, clearinghouses, open source models and liability regimes (2009).

156 Esther van Zimmeren, Sven Vanneste, Gert Matthijs, and Geertrui van Overwalle, *Patent Pools and Clearinghouses in the Life Sciences,* 29 Genet. Testing Trends Biotechnol. 569 (2011).

157 David Olson & Fabrizio Ducci, Patenting Genetic Information, 98 Indiana L. J. 1181 (2023).

158
Secretary’s Advisory Committee on Genetics, Health, and Society, Patents and Licensing Practices and Their Impact on Patient Access to Genetic Tests, *supra* note at 48.

159
*Id.* at C-4.

160
*See* Donald Zuhn, *Bio Comes Out Swinging Against SACGHS Report,* Patent Docs (Feb. 4, 2010), http://www.patentdocs.org/2010/02/bio-comes-out-swinging-against-sacghs-report.html (accessed Mar., 2024).

161
*Brief for Amicus Curiae Christopher M. Holman and Robert Cook-Deegan in Support of Neither Party, Ass’n for Molecular Pathology v. Myriad Genetics, Inc.* (Fed. Circ. No. 2010-1406) at 14–24 (2010).

## Notes

This is one of several thematically linked articles submitted for a ‘special issue’ of the *Journal of Law and the Biosciences*, resulting from the NCI-funded Sulston Project at Arizona State University and Baylor College of Medicine.

